# EZH2-mediated H3K27me3 links microbial inosine loss to depression: a gut-brain epigenetic switch

**DOI:** 10.7150/thno.120824

**Published:** 2025-09-12

**Authors:** Sen Zhu, Xuan Li, Ying Yu, Xiaoyi Han, Fang Yang, Mengxi Lu, Gaole Dai, Liang Guo, Dan Xu

**Affiliations:** 1 Department of Obstetrics, Zhongnan Hospital of Wuhan University, School of Pharmaceutical Sciences, Wuhan University, Wuhan, 430071, China.; 2 Department of Plastic Surgery, Zhongnan Hospital of Wuhan University, Wuhan University, Wuhan, 430071, China.; 3 Department of Neurology, Renmin Hospital of Wuhan University, Wuhan, 430060, China.; 4 Hubei Provincial Key Laboratory of Developmentally Originated Disease, Wuhan, 430071, China.; 5 Ministry of Education Key Laboratory of Combinatorial Biosynthesis and Drug Discovery, Wuhan, 430071, China.

**Keywords:** EZH2, gut microbes, H3K27me3, depression, coumaric acid

## Abstract

**Background:** Depression, the second most prevalent neurological disorder globally, affects over 300 million people and presents an urgent public health challenge. While gut microbiota dysbiosis is increasingly recognized as a key contributor to depression, the molecular mechanisms linking microbial imbalance to brain dysfunction remain poorly defined.

**Methods:** We investigated the role of EZH2 in gut microbiota-induced depressive behaviors in mice using the chronic unpredictable mild stress (CUMS), fecal microbiota transplantation, and conditional knockout of EZH2. CUT&Tag sequencing was employed to analyze EZH2-mediated H3K27me3 epigenetic reprogramming. Untargeted metabolomics and luciferase reporter assays were used to identify metabolites that upregulate EZH2 expression. 16S rRNA sequencing combined with metabolic tracing was conducted to trace the microbial origin of inosine. Additionally, natural compound screening identified coumaric acid (CA) as a novel EZH2-targeting degrader.

**Results:** Conditional knockout of neuronal *Ezh2* abolishes microbiota-induced depressive behaviors and neuronal apoptosis. Mechanistically, reduced abundance of specific microbiota (*f_Lachnospiraceae, f_Oscillospiraceae, and f_Erysipelotricaceae*) leads to inosine depletion. This depletion subsequently elevates EZH2 transcriptional activity by increasing H3K9ac modification at its locus, mediated through attenuation of the A2aR-cAMP-PKA-CREB-HDAC3 signaling axis. Subsequently, EZH2 silences serotonergic synapse-related genes (e.g., *Tph2, Htr2a, Htr6*) *via* H3K27me3 reprogramming, ultimately driving depressive behaviors and neuronal apoptosis in mice. Importantly, CA is identified as a first-in-class EZH2 degrader that binds lysine residues K623/K646 and recruits UBE3A for proteasomal degradation. CA treatment restores synaptic integrity and reverses depressive behaviors with minimal toxicity.

**Conclusions:** Collectively, these findings define a novel "microbiota-inosine-EZH2" axis in depression pathogenesis and highlight EZH2 degradation as a promising therapeutic strategy for microbiota-associated neuropsychiatric disorders.

## Introduction

Major depressive disorder (MDD) ranks as the fourth most prevalent disease globally and the second most common neurological disorder. It is characterized by persistent sadness, hopelessness, and feelings of worthlessness 1, 2. World Health Organization data indicates over 300 million individuals worldwide suffer from MDD 3. The condition can impair concentration and memory, posing life-threatening risks in severe cases 4. As a neuropsychiatric disorder rooted in neuronal dysfunction, current treatments (pharmacotherapy, psychotherapy, electroconvulsive therapy) are only effective for a subset of patients 5. The incomplete understanding of its pathophysiology underscores the urgent need to explore MDD mechanisms.

The pathogenesis of MDD arises from the interplay of genetic, environmental, psychological, and biological factors 6, 7. Its neuropathology involves multiple key brain regions, including the prefrontal cortex (PFC), hippocampus, amygdala, and nucleus accumbens 8, 9. Among these, the PFC plays a particularly pivotal role in MDD. Positron emission tomography studies have demonstrated a marked increase in the distribution of non-serotonergic monoamine-metabolizing enzymes within the PFC of patients with MDD 10. Notably, both the structure and function of the PFC can undergo significant changes following effective interventions, such as antidepressant therapy 11, 12. In clinical settings, treatments targeting the PFC have been shown to markedly alleviate depressive symptoms as well as comorbid somatic complaints 13. Consistently, rodent studies further support the central role of the PFC in mediating depression-like behaviors 14-16.

Recent evidence links depression to gut microbiota dysbiosis 17. MDD patients exhibit significantly altered microbiota composition compared to healthy individuals. Fecal microbiota transplantation (FMT) from depressed patients to germ-free mice induces depressive-like behaviors and neuronal damage. Similarly, depressed mice show altered gut microbiota, and their FMT can transfer depressive behaviors 18, 19. Studies further demonstrate that modulating gut microbiota or supplementing its metabolites alleviates depressive symptoms in mice 20-28. Despite highlighting the microbiota's role, the underlying mechanisms remain unclear.

Neuronal regenerative capacity declines with maturation 29. During development, gene repression in neurons is primarily mediated by polycomb repressive complex 2 (PRC2) through histone methyltransferases EZH1 and EZH2 30. EZH2 silences gene expression via H3K27me3 catalysis. While its expression decreases during synaptic maturation, EZH2 persists in adult neurons, indicating ongoing PRC2-mediated silencing in the mature brain 31. Notably, elevated EZH2 levels are reported in the brains of depressed mice and correlate strongly with depressive-like behaviors 32-34, suggesting EZH2 may be a key mediator in MDD pathogenesis.

In this study, we used the chronic unpredictable mild stress (CUMS) model, FMT, and neuronal EZH2 conditional knockout to investigate EZH2's role in gut microbiota-induced depressive behaviors and neuronal apoptosis in mice. Mechanistically, we employed CUT&Tag sequencing to analyze EZH2-mediated H3K27me3 reprogramming. Untargeted metabolomics and in vitro experiments identified Inosine depletion as a key event driving EZH2 upregulation. 16S rRNA sequencing and metabolite tracing linked reduced Inosine levels to decreased abundance of specific microbiota (*f_Lachnospiraceae*, *f_Oscillospiraceae*, *f_Erysipelotrichaceae*). Finally, natural product screening identified the EZH2-targeted inhibitor coumaric acid (CA), which effectively alleviated depressive behaviors and neuronal apoptosis in mice. This study advances our understanding of MDD pathogenesis and identifies potential therapeutic candidates.

## Materials and Methods

### Animals

C57BL/6 female mice (18-23 g) were purchased from Shanghai Nomo Biotechnology Co., Ltd. Mice were housed under standard conditions with controlled temperature and a 12-hour light/dark cycle, with free access to food and water before experiments.

### Animal experimental treatment

CUMS and FMT procedure: Mice were divided into control and CUMS groups (n=15). CUMS mice were exposed to two random stressors daily for five consecutive weeks, including 45° cage tilting (24 h), food deprivation (24 h), water deprivation (24 h), wet bedding (24 h), swimming in 4 °C water (5 min), rotating at 120 rpm (30 min), and restraint in a 50 mL tube (6 h). Control mice were housed under normal conditions. After CUMS, mouse fecal samples were collected and used for subsequent FMT experiments 35. Specifically, fresh fecal pellets were collected from donor mice and immediately diluted in sterile phosphate-buffered saline (PBS). One milliliter of PBS was used to dilute 50 mg of fecal material. The fecal samples were soaked in sterile PBS for approximately 15 minutes, vortexed thoroughly, and centrifuged at 1,000 rpm for 5 minutes at 4 °C. The supernatant was centrifuged again at 8,000 rpm for 5 minutes at 4 °C. The resulting supernatant was discarded, and the bacterial pellet was resuspended in PBS and filtered twice. For two consecutive weeks, 100 μL of the bacterial suspension (1×10^8^ CFU/mL) was administered to antibiotic-cleared pseudo-germ-free recipient mice (ABX mice) *via* oral gavage. Mice receiving fecal microbiota from control donors were designated as the FMT-Control group, whereas those receiving fecal microbiota from CUMS donors were designated as the FMT-CUMS group. These mice were subsequently used for downstream analyses. ABX mice were generated by orally gavaging a mixture of antibiotics (neomycin 1 mg/mL, ampicillin 1 mg/mL, metronidazole 0.5 mg/mL, and vancomycin 0.5 mg/mL) for two consecutive weeks. The details of antibiotic purchases are listed in Table S1 (Supplementary materials).

EZH2 knockout experiment: EZH2^flox/flox^ mice were crossed with Syn-CreER mice to generate F1 Syn-CreER: EZH2^flox/-^ mice. F1 mice were then bred and neuron-specific EZH2 conditional knockout mice were obtained by tamoxifen (100mg/kg, HY-13757A, MedChemExpress, USA) induction (Syn-CreER: EZH2^-/-^, cKO-EZH2). cKO-EZH2 mice were obtained from Cyagen Biosciences Inc (Suzhou, China). Antibiotics were administered to EZH2^flox/flox^ and EZH2^-/-^ mice to generate ABX mice. Mice were divided into three groups (n=12): (1) FMT-Control + EZH2^flox/flox^, (2) FMT-CUMS + EZH2^flox/flox^, and (3) FMT-CUMS + EZH2^-/-^. The FMT procedure followed the previously described protocol.

Tph2* in vivo* expression experiments: Using a stereotaxic instrument, AAV9-FEV-Control and AAV9-FEV-Tph2 were injected into the PFC of FMT-Control and FMT-CUMS mice. After injection, mice were returned to their cages for a five-day recovery period, followed by behavioral analysis and euthanasia for tissue collection. Mice were divided into four groups (n=14 per group): (1) FMT-Control + AAV9-FEV-Control, (2) FMT-Control + AAV9-FEV-Tph2, (3) FMT-CUMS + AAV9-FEV-Control, and (4) FMT-CUMS + AAV9-FEV-Tph2. AAV9-FEV-Control and AAV9-FEV-Tph2 were constructed and purchased from Gemma Bio Inc (Shanghai, China). Titer 5E+12 vg/ml, injection volume 100 nL, injection rate 20 nL/min. Mice were anesthetized with isoflurane during the operation.

Inosine supplementation experiments: for the inosine treatment, 50 mg/kg/day of inosine (HY-N0092, MedChemExpress, USA) dissolved in saline was supplemented to mice by intraperitoneal injection for two weeks.

*R. hominis* gavage administration: *R. hominis* was obtained from Wuhan Zhengbei Technology Co., Ltd (Wuhan China). The bacteria were cultured overnight under anaerobic conditions in modified YCFA medium (Hopebio, Qingdao, China,) supplemented with 0.2% glucose, maltose, and cellobiose (Selleck chemicals, S4704, China). The cultures were centrifuged at 5000 rpm for 30 minutes, and the supernatant was collected. The bacterial pellet was resuspended in sterile PBS and administered to mice by oral gavage at a dose of 1 × 10⁹ CFU per mouse daily for two consecutive weeks.

CA *in vivo* intervention experiment: CA (HY-32004, MedChemExpress, USA) was administered (40 mg/kg) via intraperitoneal injection to FMT-CUMS mice once daily for two weeks. After the intervention, behavioral analysis was performed, followed by euthanasia for tissue collection. Mice were divided into four groups (n=12): (1) FMT-Control, (2) FMT-CUMS, and (3) FMT-CUMS + CA.

### CUT&Tag analysis of H3K27me3 changes

Briefly, 100,000 cells were harvested, centrifuged at 600×g for 3 min at room temperature, and washed twice with 300 μL Wash Buffer. Cells were incubated with 10 μL concanavalin A-coated magnetic beads at RT for 10 min, then the unbound supernatant was removed. Bead-bound cells were resuspended in 50 μL Dig-wash Buffer containing 2mM EDTA and a 1:50 dilution of the primary antibody (H3K27me3) and incubated on a rotating platform for 2 h at RT. After removing the liquid, a 1:50 diluted secondary antibody (IgG) was added in 50 μL Dig-Wash Buffer and incubated at RT for 1 h; for the IgG control library, only the secondary antibody was used. Cells were washed twice with 500 μL Dig-Wash Buffer for 1 min using a magnetic stand. A 1:200 dilution of pG-Tn5 (~0.04 μM) was prepared in Dig-300 Buffer and added to the cells with gentle vortexing, followed by incubation at RT for 1 h. Cells were washed twice with 500 μL Dig-300 Buffer and resuspended in 300 μL Tagmentation Buffer, then incubated at 37 °C for 1 h. The reaction was stopped by adding 10 μL 0.5M EDTA, 3 μL 10% SDS, and 2.5 μL 20 mg/mL Proteinase K, followed by incubation at 55 °C for 1 h. DNA was purified using phenol-chloroform-isoamyl alcohol extraction, ethanol-washed, and resuspended in water. DNA amplification was performed by PCR with an initial step of 72 °C for 3 min and 98 °C for 30 s, followed by 16 cycles of 98 °C for 15 s, 60 °C for 30 s, and 72 °C for 30 s, with a final extension at 72 °C for 3 min. The amplified DNA was purified using Ampure XP beads and sequenced on an Illumina NovaSeq platform (2×150 nt). The CUT&Tag experiments were performed by ZhenYue Bioinformatics Co., Ltd.

### Golgi stain analysis

Intact brain tissues were fixed in Golgi staining fixative (Servicebio, China), then immersed in Golgi staining solution for 14 days, with the solution replaced every 3 days. After staining, tissues were sectioned at 5-10 mm thickness using a vibrating microtome (Leica VT1000S). The sections were treated with tissue treatment solution and imaged.

### Cellular thermal shift assay

HeLa cells were treated with CA for 48 hours, followed by cell collection and protein extraction. *In vitro*, the extracted proteins were subjected to a temperature gradient heating at 35, 40, 45, 50, 55, 60, 65, 70, 75, and 80 °C. Protein levels were then analyzed by Western blotting. The Ezh2-K623R mutant plasmid was custom synthesized by MiaolingBio Co., Ltd.

### Plasmid construction and RNA interference

Briefly, shRNA sequences were designed through the website provided by Merck and cloned into the pLKO.1-shRNA vector. It was transformed into DH5α, identified by sequencing, amplified, and extracted for cell transfection. The sequences were synthesized by China Tianyi Company, Inc. Plasmid sequence design and construction are referenced to previous studies. shRNA sequence reference table S4 (Supplementary materials).

### Cell viability analysis

The HT22 cells were treated with CA for 12 h, 24 h and 48 h, and then cell viability was assayed by CCK8 assay.

### Study approval

All behavioral tests were conducted by the same experimenter, blinded to group assignments, to minimize inter-experimenter variability. This study followed the NIH Guide for the Care and Use of Laboratory Animals and was approved by the Animal Ethics Committee of Wuhan University.

### Data analysis and statistics

For data analysis, statistical significance was determined using appropriate tests, such as Student's t-test for two-group comparisons or one-way ANOVA for multiple comparisons. All data are presented as mean ± standard error of the mean (SEM). P-values less than 0.05 were considered statistically significant. Data were analyzed using software such as GraphPad Prism or Origin.

Additional experimental materials and methods are provided in the **Supplementary Information**.

## Results

### Gut microbiota mediates the upregulation of EZH2 levels in the brains of depressed mice

**Figure 1:**
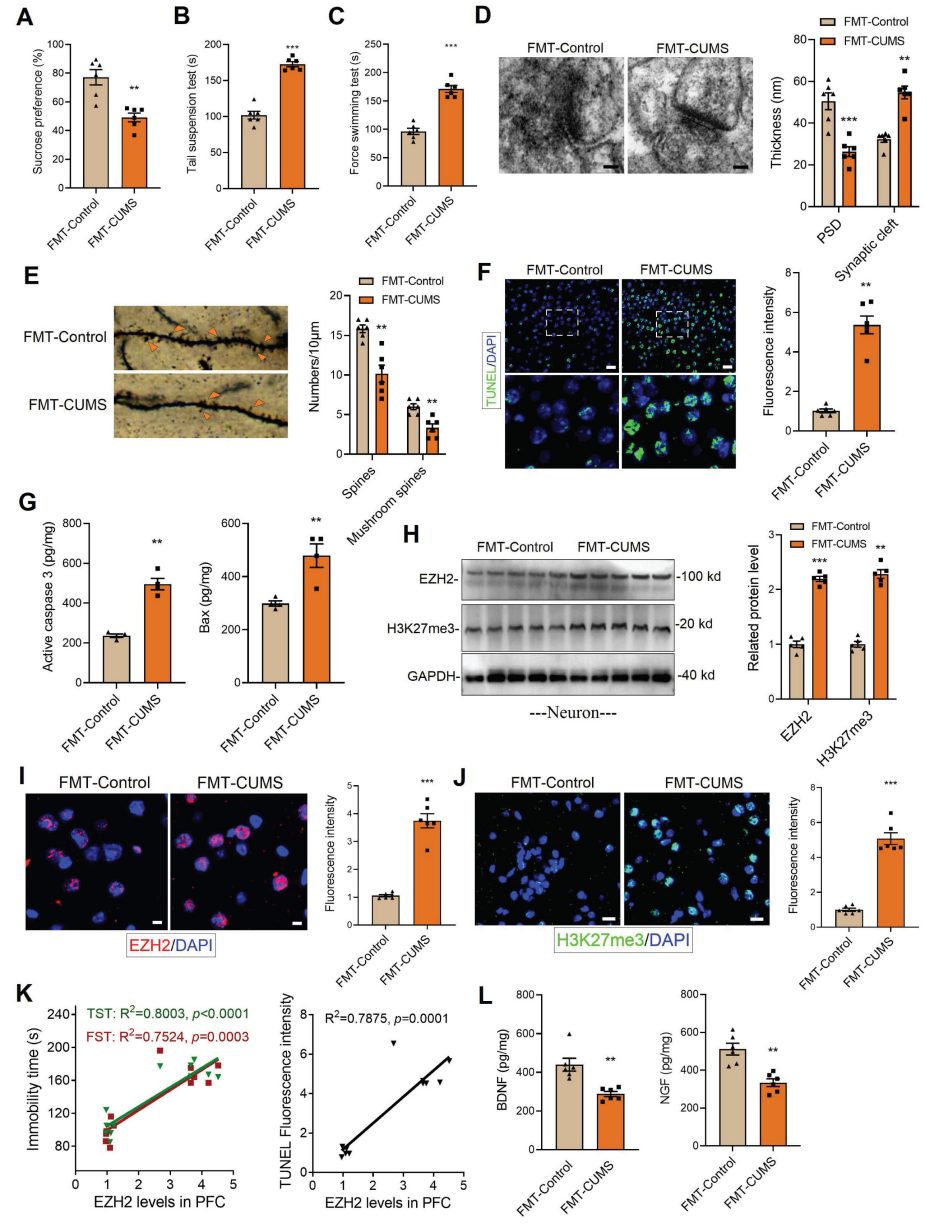
Increased EZH2 and H3K27me3 levels in the PFC of FMT-CUMS mice. (A-C) Behavioral tests assessing depressive-like behaviors in mice, including the FST, TST and SPT. (D) TEM showing synaptic ultrastructure. (E) Golgi staining analysis of synaptic structures. (F) TUNEL staining to assess neuronal apoptosis in the PFC. (G) ELISA analysis of activated caspase 3 and Bax protein levels. (H) Western blot analysis of EZH2 and H3K27me3 levels in Primary neuron. (I-J) Immunofluorescence staining of EZH2 and H3K27me3 in the PFC. (K) Correlation analysis between EZH2 expression, depressive behaviors, and neuronal apoptosis. (L) Quantification of BDNF and NGF levels in the PFC. * vs. FMT-Control, *p < 0.05, **p < 0.01, ***p < 0.001.

Following established methods, we induced a depression model in mice using the CUMS paradigm. ABX mice were then gavaged with fecal suspensions from either control or CUMS mice, designated as FMT-Control and FMT-CUMS, respectively (Figure S1A). Depressive behaviors were assessed using the sucrose preference test (SPT), tail suspension test (TST), and forced swimming test (FST). The results showed that FMT-CUMS mice had a significantly reduced preference for sucrose and spent significantly more time immobile in the TST and FST (Figure 1A-C). The prefrontal cortex (PFC) plays a crucial role in emotional regulation, social behavior, and self-control. In patients with MDD, the PFC is among the most severely impaired brain regions 36. Therefore, we selected the PFC as the target region for further investigation. We further analyzed two neurotrophic factors, BDNF and NGF, and found that their levels were significantly lower in FMT-CUMS mice (Figure 1L and S1B). Transmission electron microscopy (TEM) and Golgi staining were used to assess the ultrastructure of neuronal synapses. The results revealed that FMT-CUMS mice exhibited an increased synaptic cleft width, reduced postsynaptic membrane thickness, decreased dendritic spine density and branching, and shortened dendritic length (Figure 1D-E and S1C). In addition, we analyzed the levels of the presynaptic membrane marker protein MRX96 and the postsynaptic membrane marker protein PSD95 and found that the levels of both proteins were significantly reduced in the PFC of FMT-CUMS mice (Figure S1D). Neuronal apoptosis in the brain has also been recognized as one of the fundamental mechanisms underlying depression. Therefore, we further examined apoptosis in the PFC of CUMS mice. The results showed an increase in the number of TUNEL-positive cells in the PFC (Figure 1F). In addition, ELISA analysis of apoptosis-related proteins indicated an upregulation of activated caspase-3 and Bax levels (Figure 1G).

Further, we analyzed changes in EZH2 expression in the PFC of mice. The results showed that the protein levels of EZH2 in the PFC of FMT-CUMS mice were higher than those in the FMT-Control mice (Figure 1I). As a key histone methyltransferase, EZH2 catalyzes H3K27me3. We further examined changes in H3K27me3 levels and observed a similar increase in H3K27me3 (Figure 1J). Additionally, we performed a correlation analysis between EZH2 levels and depressive behaviors as well as neuronal apoptosis in mice and found that EZH2 expression was positively correlated with both the severity of depression and the extent of apoptosis. (Figure 1K). In summary, these findings suggest that EZH2 may serve as a key target in the pathogenesis of depression and is regulated by gut microbiota.

### Neuronal conditional knockout of EZH2 alleviates gut microbiota-mediated depressive behaviors and neuronal apoptosis

Neurons, microglia, and astrocytes are the most abundant cell types in the brain. In CUMS mice, microglia are activated, with their numbers remaining unchanged 18, 22. Extensive evidence suggests that neuronal dysfunction is considered a direct factor triggering neurobehavioral abnormalities. To further investigate, we isolated neurons, microglia, and astrocytes from the PFC of mice and examined EZH2 levels. The results showed that EZH2 was specifically upregulated in neurons of FMT-CUMS mice, while its expression remained unchanged in microglia and astrocytes (Figure 1H and S2A-B). This suggests that gut microbiota may specifically upregulate neuronal EZH2, mediating behavioral deficits and neuronal apoptosis in mice.

**Figure 2:**
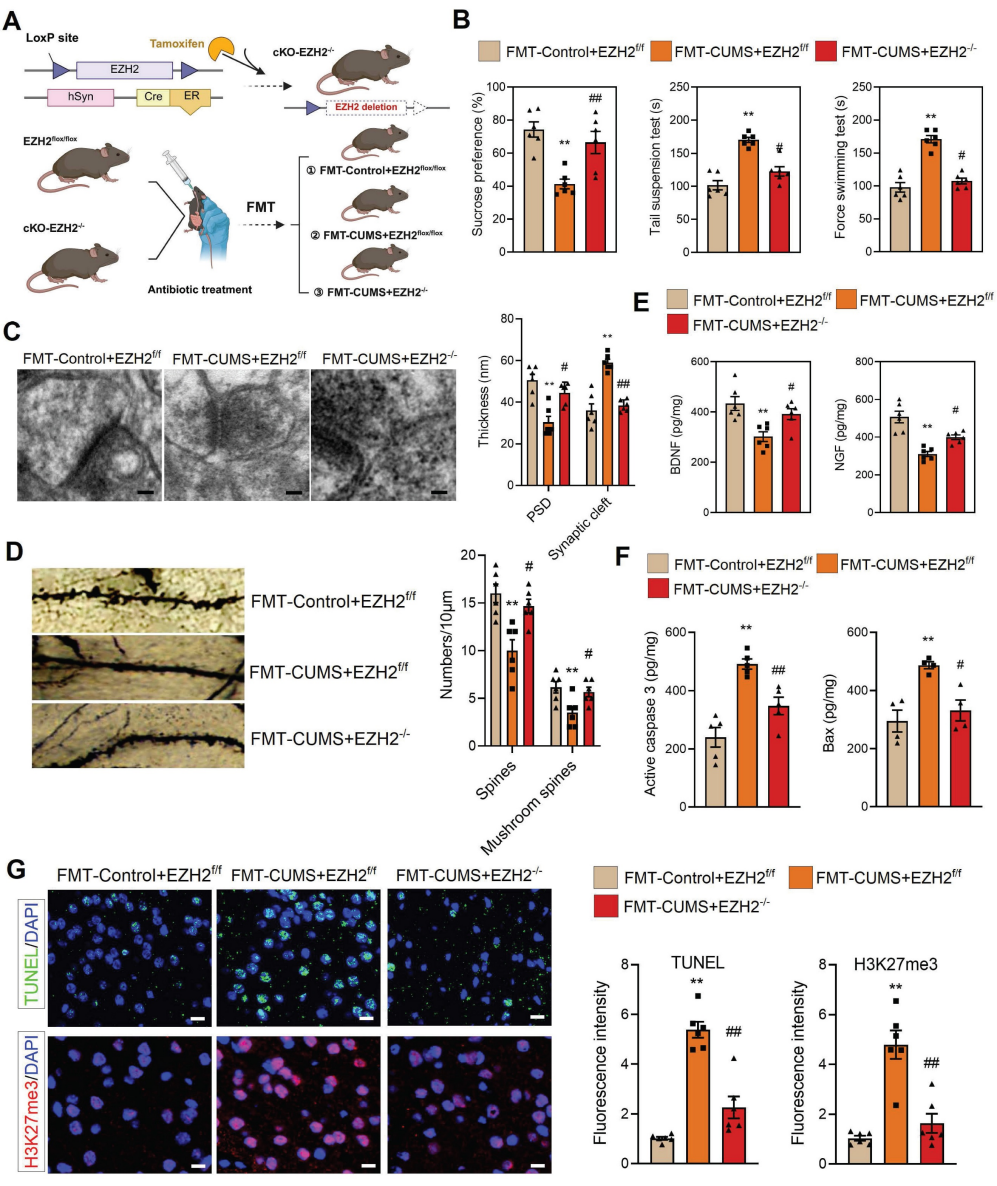
Neuronal EZH2 knockout alleviates depressive behaviors and reduces neuronal apoptosis in FMT-CUMS mice. (A) Flowchart of EZH2 Conditional Knockdown and FMT. (B) FST, TST and SPT assessment of depressive behaviors. (C) TEM images showing synaptic ultrastructure. (D) Golgi staining analysis of synaptic structures. (E) Quantification of BDNF and NGF levels in the PFC. (F) ELISA analysis of activated caspase 3 and Bax protein levels. (G) TUNEL staining for apoptotic cell detection. Immunofluorescence staining of H3K27me3 in the PFC. * vs. FMT-Control+EZH2^f/f^, *p < 0.05, **p < 0.01; ^#^ vs. FMT-CUMS+EZH2^f/f^, *^#^p <* 0.05, *^##^p <* 0.01.

To verify this, we crossed hSyn-CreER mice with EZH2^flox/flox^ mice and induced recombination via tamoxifen administration to generate neuron-specific EZH2 conditional knockout mice (cKO-EZH2 mice) (Figure 2A). Subsequently, we subjected cKO-EZH2 mice to antibiotic treatment and FMT, and analyzed depressive behaviors, synaptic alterations, and neuronal apoptosis. The results showed that cKO-EZH2 mice exhibited an increased preference for sucrose and decreased immobility time in both the TST and FST (Figure 2B). Golgi staining and TEM revealed that cKO-EZH2 mice displayed a reduced synaptic cleft width, restored postsynaptic membrane thickness, and increased dendritic spine density and branching (Figure 2C-D). Additionally, the levels of NGF and BDNF were significantly restored (Figure 2E). Furthermore, we assessed the effect of EZH2 knockout on neuronal apoptosis and found that cKO-EZH2 mice had fewer TUNEL-positive cells in the PFC, along with reduced levels of activated caspase-3 and Bax (Figure 2F-G). These findings suggest that elevated neuronal EZH2 levels mediate depressive behaviors, synaptic damage, and neuronal apoptosis in FMT-CUMS mice. Notably, we found that EZH2 knockout reversed the increase in H3K27me3 levels in the PFC of FMT-CUMS mice, suggesting that EZH2 may exert its detrimental effects through the regulation of H3K27me3 modifications (Figure 2G).

Notably, gut microbiota has also been implicated in mediating anxiety-like behaviors in mice 37-39. Consistent with previous reports, FMT-CUMS mice exhibited anxiety-like phenotypes in the open field test, marble burying test, and elevated plus maze. However, neuronal-specific EZH2 knockout exerted only minimal effects on these gut microbiota—induced anxiety-like behaviors: it partially reduced the number of buried marbles in the marble burying test but failed to significantly improve performance in the open field or elevated plus maze tests (Figure S3A-B). These findings further indicate that elevated neuronal EZH2 levels are primarily involved in gut microbiota-mediated depressive behaviors, rather than anxiety-like behaviors, in mice.

### EZH2 reprogramming H3K27me3 silences serotonergic synaptic genes

**Figure 3:**
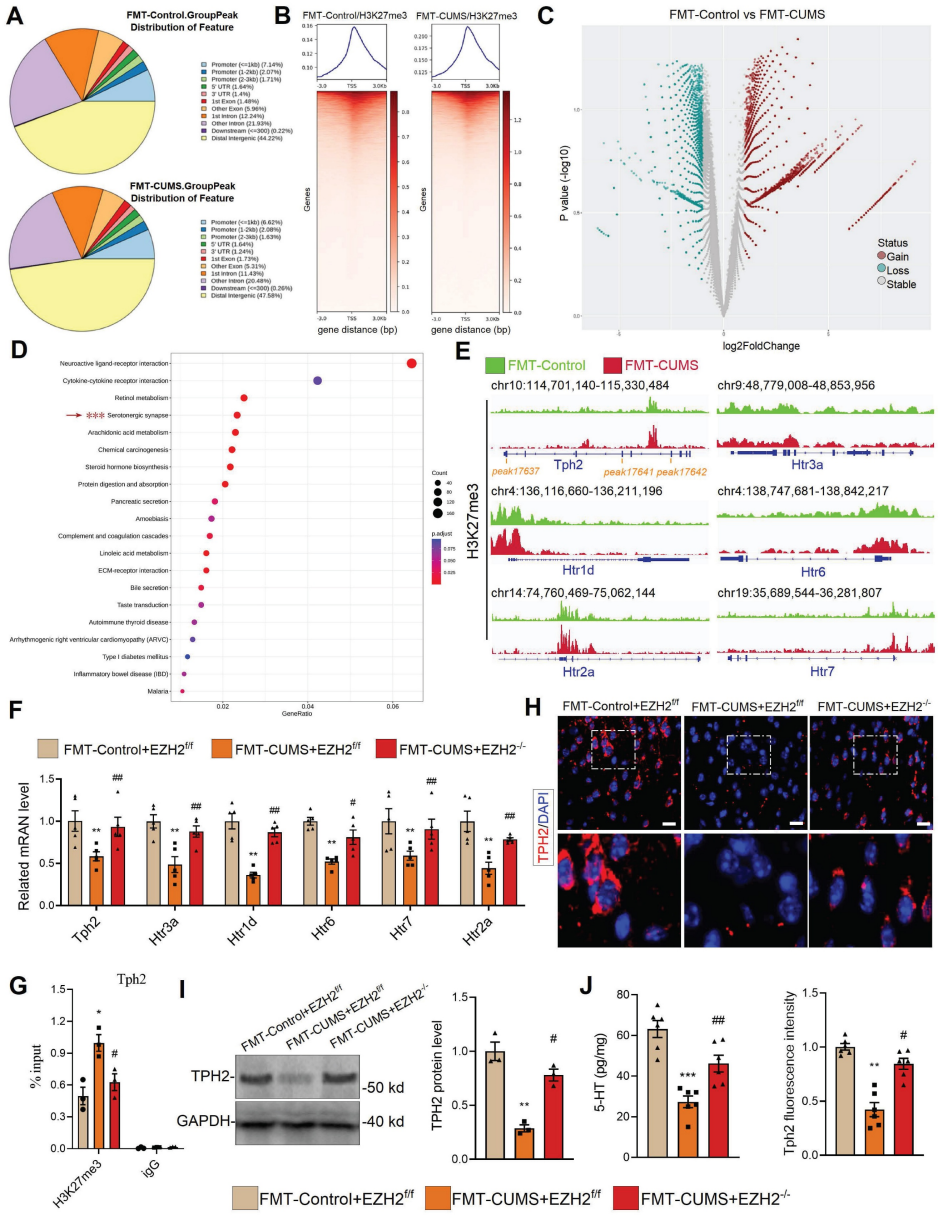
EZH2-mediated H3K27me3 reprogramming suppresses serotonergic synapse function. (A) Pie chart displaying differential H3K27me3-modified regions. (B) Heatmap showing H3K27me3 enrichment at TSS. (C) Volcano map of differential H3K27me3-modified peaks. (D) KEGG pathway analysis of differential H3K27me3-modified genes. (E) Chromatin landscape of selected differential H3K27me3 peaks. (F) RT-qPCR analysis of *Tph2, Htr3a, Htr1d, Htr6, Htr7,* and *Htr2a* mRNA expression. (G) CUT&Tag-qPCR analysis of H3K27me3 occupancy at the *Tph2* locus. (H) Immunofluorescence analysis of TPH2. (I) Western blot analysis of TPH2 protein levels. (J) ELISA quantification of 5-HT levels in PFC. * vs. FMT-Control+EZH2^f/f^, *p < 0.05, **p < 0.01; ^#^ vs. FMT-CUMS+EZH2^f/f^, *^#^p <* 0.05, *^##^p <* 0.01.

As a core subunit of PRC2, EZH2 catalyzes the H3K27me3 modification to silence gene expression. We hypothesized that the depressive behaviors and neuronal apoptosis induced by EZH2 in mice may also result from changes in the H3K27me3 pattern. We performed CUT&Tag-seq on the PFC tissues of FMT-Control and FMT-CUMS mice. Approximately 10% of H3K27me3-enriched peaks overlapped with promoter regions, while more than half were located in intergenic regions (Figure 3A-B), consistent with previously reported patterns of H3K27me3 modification 40. The results showed that, compared to the FMT-Control group, FMT-CUMS mice exhibited a significant increase in H3K27me3 levels in 34,166 peaks, while 11,425 peaks had significantly decreased H3K27me3 levels (Figure 3C). Cellular component enrichment analysis revealed that the differential peaks were predominantly enriched in postsynaptic membrane components, postsynaptic density, asymmetric synapses, and ion channel-related components, suggesting that gut microbiota of depressed mice indeed exerts influence on neuronal function (Figure S3C). Moreover, KEGG analysis showed that in FMT-CUMS mice, neuroactive ligand-receptor interactions, cytokine-cytokine receptor interactions, retinol metabolism, serotonergic synapses, and arachidonic acid metabolism pathways had high H3K27me3 modifications, suggesting that these signals received inhibition (Figure 3D). Among these, serotonergic neuron dysfunction is considered to be a key mechanism mediating depression, particularly deficient 5-HT synthesis and reuptake disorders 41. Then we analyzed the H3K27me3 levels of genes enriched in serotonergic synapses, including tryptophan hydroxylase 2 (*Tph2), 5-Hydroxytryptamine receptor (Htr) 1d (Htr1d), Htr2a, Htr3a, Htr5,* and *Htr7*. All of these genes exhibited high levels of H3K27me3 modification (Figure 3E). Further examination of these genes revealed a significant decrease in the mRNA levels of *Tph2, Htr1d, Htr2a, Htr3a, Htr5,* and *Htr7* in the PFC of FMT-CUMS mice (Figure 3F). Notably, EZH2 knockout recovered the expression of these genes (Figure 3F). Moreover, through CUT&Tag-qPCR analysis, we found that EZH2 knockout reduced the H3K27me3 levels of these genes (Figure 3G and S3D).

TPH2, as the key rate-limiting enzyme in serotonergic neurons, catalyzes the conversion of tryptophan to 5-hydroxytryptophan. Disruption or mutation of *Tph2*, leading to impaired serotonin synthesis, is considered a typical mechanism underlying the onset of depression 42. Pharmacological activation of TPH2 to promote serotonin synthesis is a key therapeutic strategy for improving depression. Further, we assessed the protein levels of TPH2 and the content of 5-HT. The results showed a decrease in TPH2 protein levels and impaired 5-HT synthesis in the PFC of FMT-CUMS mice (Figure 3H-J). Notably, EZH2 knockout was able to restore TPH2 levels and 5-HT synthesis (Figure 3H-J). These results confirm that EZH2 inhibits 5-HT synthesis by silencing *Tph2* transcription through H3K27me3.

### TPH2 re-expression improved 5-HT synthesis and alleviated depressive behaviors in FMT-CUMS mice

**Figure 4:**
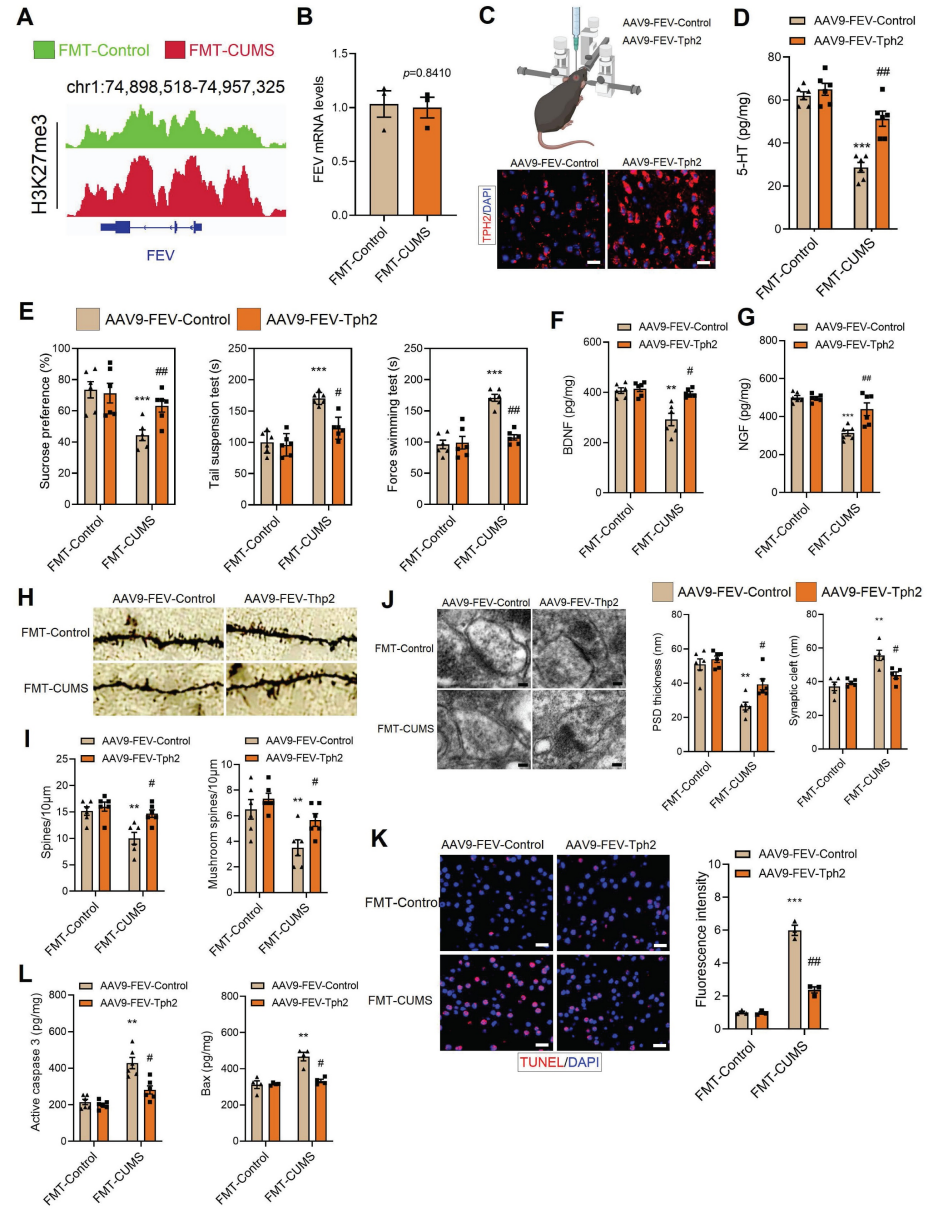
TPH2 re-expression restores 5-HT synthesis and alleviates depressive behaviors in FMT-CUMS mice. (A) Chromatin landscape of H3K27me3 modification at the FEV locus. (B) RT-qPCR analysis of *Fev* mRNA expression. (C) Schematic representation of AAV injection into the PFC. (D) ELISA quantification of 5-HT levels in the PFC. (E) SPT, TST and FST assess depressive behaviors. (F-G) BDNF and NGF levels in the PFC. (H-I) Golgi staining analysis of synaptic structures. (J) TEM images of synaptic ultrastructure. (K) TUNEL staining for neuronal apoptosis assessment. (L) ELISA analysis of activated caspase 3 and Bax protein levels. * vs. FMT-Control, *p < 0.05, **p < 0.01; ^#^ vs. AAV9-FEV-Tph2, *^#^p <* 0.05, *^##^p <* 0.01.

To confirm that *Tph2* transcriptional repression is a key mediator of depressive behaviors, we re-expressed *Tph2* in serotonergic neurons. In FMT-CUMS mice, EZH2 reprogramming of H3K27me3 inhibits *Tph2* transcription. Therefore, we used an alternative serotonergic neuron-specific promoter, FVE, which is unaffected, to express *Tph2* (Figure 4A-B). We injected AAV9-FVE-Control and AAV9-FVE-Tph2 into the PFC of mice to achieve TPH2 expression (Figure 4C). We found that TPH2 re-expression restored 5-HT synthesis and increased sucrose preference in FMT-CUMS mice, as well as shortened the immobility time in both the TST and FST (Figure 4D-E). The levels of BDNF and NGF were also partially restored (Figure 4F-G). Additionally, we observed that TPH2 re-expression reduced the synaptic cleft width, increased postsynaptic membrane thickness, dendritic spine density, and dendritic branching (Figure 4H-J). Moreover, TPH2 re-expression reduced the number of TUNEL-positive cells in the PFC of FMT-CUMS mice and decreased the expression of activated caspase-3 and Bax (Figure 4K-L).

### Gut microbiota-driven reduction of inosine upregulates EZH2 expression

**Figure 5:**
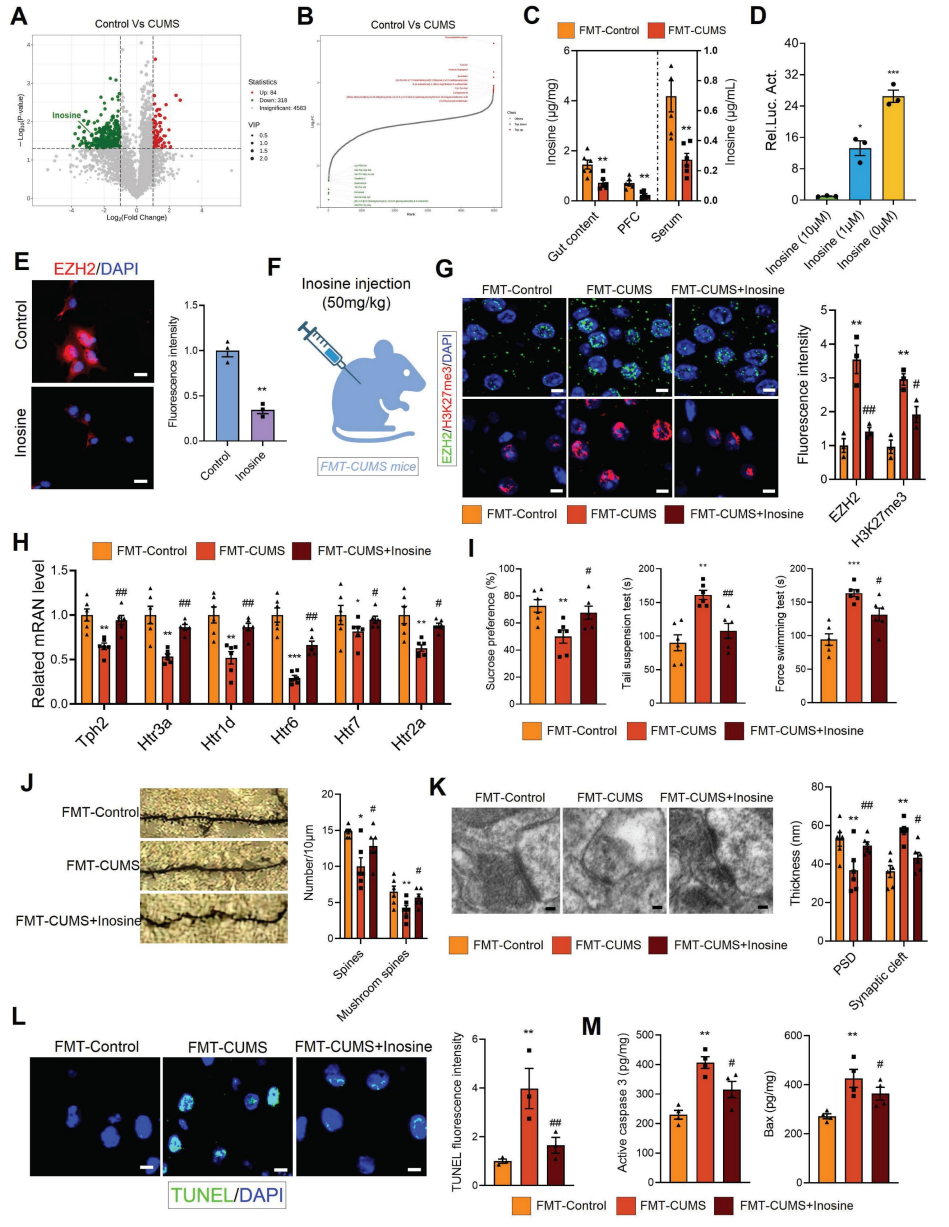
Gut microbiota-driven inosine reduction induces EZH2 transcriptional activation and neurobehavioral deficits. (A-B) Volcano plot and distribution of differential fecal metabolites. (C) Mass spectrometry quantification of inosine levels in the gut content, serum and PFC. (D) Luciferase reporter assay analysis of inosine's effect on *Ezh2*. (E) Immunofluorescence analysis of EZH2 in cells. (F) Schematic diagram of inosine supplementation. (G) Protein levels of EZH2 and H3K27me3 in the PFC. (H) RT-qPCR analysis of *Tph2, Htr3a, Htr1d, Htr6, Htr7,* and *Htr2a* mRNA expression. (I) FST, TST and SPT assessing depressive behaviors. (J) Golgi staining analysis of synaptic structures. (K) TEM images showing synaptic ultrastructure. (L) TUNEL staining for neuronal apoptosis assessment. (M) Activated caspase 3 and Bax protein levels in the PFC. * vs. Control/FMT-Control, *p < 0.05, **p < 0.01; ^#^ vs. FMT-CUMS, ^#^*p <* 0.05, *^##^p <* 0.01.

Gut microbiota regulates host health through the synthesis or secretion of its metabolic products 43. For instance, gut microbiota-derived short-chain fatty acids and homovanillic acid have been shown to significantly improve depression 21, 44. To further investigate the potential molecular basis of gut microbiota-mediated EZH2 upregulation, we performed untargeted metabolomic analysis on the fecal samples of control and CUMS mice. Orthogonal Partial least squares discriminant analysis (OPLS-DA) and principal component analysis (PCA) results showed significant differences between the groups (Figure S4A-B). Additionally, we analyzed the differential metabolites. Compared to control feces, CUMS mouse feces exhibited significant alterations, with 84 metabolites significantly upregulated and 318 metabolites significantly downregulated (LogFC > 1, p < 0.05) (Figure 5A-B). The altered metabolites were primarily enriched in heterocyclic compounds, amino acids and their derivatives, and organic acids and their derivatives (Figure S4C). KEGG analysis indicated that the enriched pathways of differential metabolites were closely related to synaptic development and neurological disorders, including synaptic vesicle cycling, serotonergic synapses, long-term depression, dopaminergic synapses, axon regeneration, and Parkinson disease (Figure S4D). Further, we performed metabolic tracing analysis of the 402 differential metabolites using MetOrigin 2.0. Several candidate metabolites of microbial origin were identified, including dulcitol, anserine, D-phenylalanine, γ-Glutamyltyramine, inosine, pueraria, pyrocatechol, and coproporphyrin III. Among them, we identified inosine, which has neuroprotective effects and is known to ameliorate various neurological disorders, including Parkinson disease, mania, and depression 45-48. We then measured inosine levels in serum, PFC and gut content using mass spectrometry, and found that all three compartments exhibited significantly reduced inosine levels in FMT-CUMS mice (Figure 5C).

To explore the relationship between inosine and EZH2, we conducted luciferase reporter assays and immunofluorescence analyses. The transcriptional level of *Ezh2* increased significantly as inosine concentration decreased, and this was further confirmed at the protein level (Figure 5D-E). Based on these results, we hypothesized that gut microbiota may activate EZH2 transcription by reducing inosine levels. To verify the role of inosine, we administered intraperitoneal injections of inosine to FMT-CUMS mice for two consecutive weeks (Figure 5F). Inosine supplementation reduced EZH2 and H3K27me3 levels in the PFC region, restoring the expression of *Tph2*, *Htr1d*, *Htr2a*, *Htr3a*, *Htr5*, and *Htr7* (Figure 5G-H). Moreover, inosine supplementation alleviated depressive behaviors, improved synaptic damage, and attenuated neuronal apoptosis in FMT-CUMS mice (Figure 5I-M).

Adenosine A2A receptor (A2aR) is a well-known receptor for inosine 49. To further investigate whether A2aR is involved in the inhibitory effect of inosine on EZH2, we treated cells *in vitro* with inosine and the shA2aR plasmid. The results showed that A2aR knockdown restored EZH2 expression (Figure S5A and S5D). As a G protein-coupled receptor, A2aR activation of the downstream cAMP-PKA-CREB signaling pathway can improve neurodegenerative disorders 50. Further, we investigated whether this signaling axis is involved in the process. The results demonstrated that either the PKA inhibitor H-89 or CREB knockdown could reverse the transcriptional inhibition of EZH2 by inosine, which was further confirmed at the protein level *via* immunofluorescence (Figure S5A and S5D).

To further understand the potential mechanism by which cAMP-PKA-CREB inhibits EZH2, we used Cistrome DB to analyze regulatory modifications associated with *Ezh2* expression. H3K9ac and H3K27ac were the two most significant modifications regulating EZH2 (Figure S5B). Additionally, PPI database analysis identified proteins interacting with CREB, among which HDAC1, HDAC2, and HDAC3 were observed (Figure S5C). Therefore, we hypothesized that inosine activates CREB to recruit histone deacetylases, thereby reducing H3K9ac or H3K27ac modifications to inhibit EZH2 transcription. Subsequent individual knockdowns of HDAC1, HDAC2, and HDAC3 revealed that only HDAC3 knockdown abolished the inhibitory effect of inosine on EZH2 transcription (Figure S5E-F). Further, CHIP-qPCR analysis was conducted *in vitro* to assess H3K9ac and H3K27ac modifications at the *Ezh2* locus. The results showed that inosine reduced H3K9ac levels, which was reversed by HDAC3 knockdown, while inosine had no effect on H3K27ac (Figure S5G). In summary, these results suggest that inosine depletion can upregulate EZH2 transcription by inhibiting the A2aR-cAMP-PKA-CREB-HDAC3 signaling pathway.

### Supplementation with *R. hominis* increased inosine levels and alleviated depressive behaviors and neuronal apoptosis

**Figure 6:**
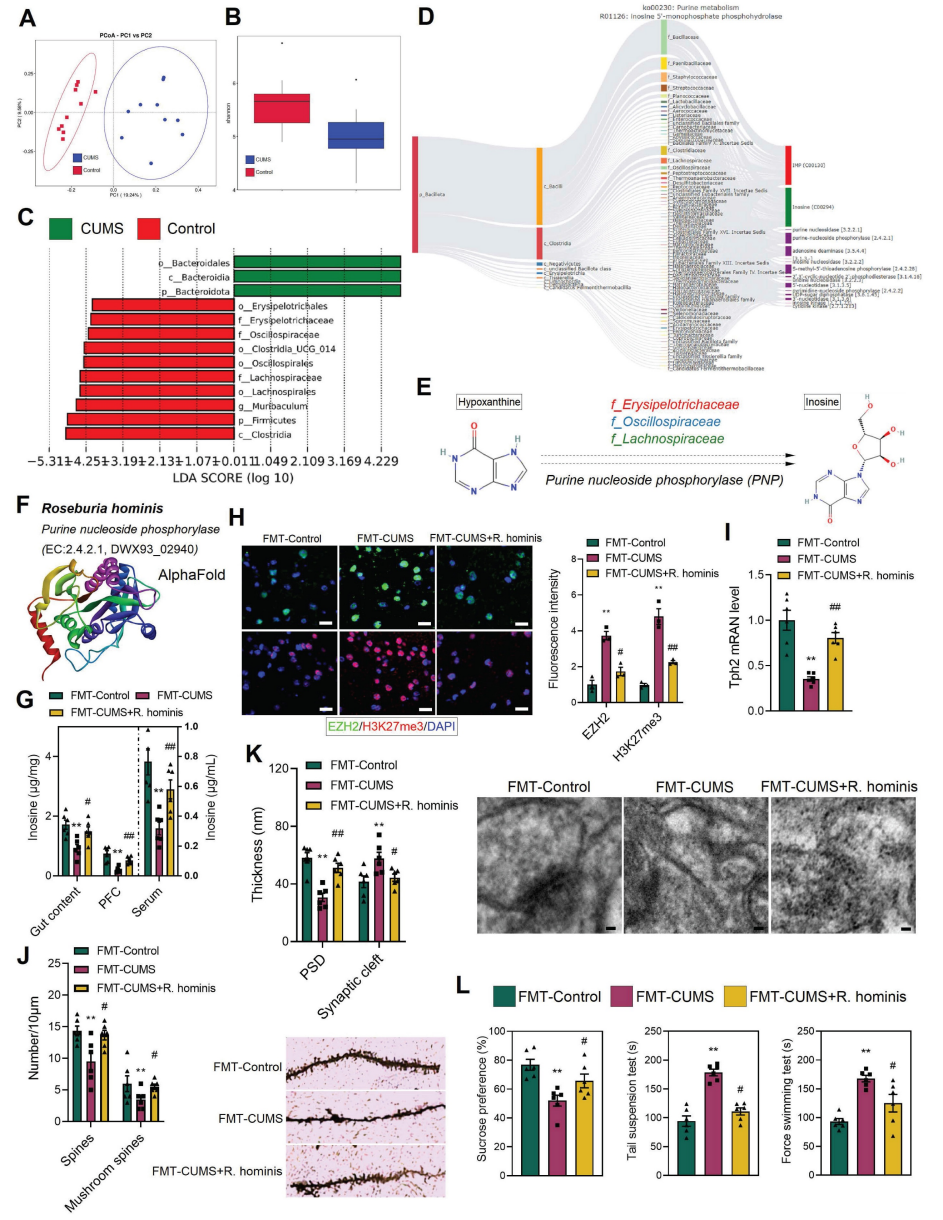
*R. hominis* supplementation increases inosine levels and alleviates gut microbiota-mediated depressive phenotypes. (A) PCoA reveals intergroup distances of gut microbiota. (B) Shannon index indicates the abundance of gut microbiota. (C) Lefse analysis of differential gut microbiota. (D) MetOrigin-based source tracking of inosine. (E) Catalytic process of hypoxanthine conversion to inosine. (F) *R. hominis* information in Uniprot. (G) Inosine levels in the gut content, serum and PFC. (H) Immunofluorescence staining of EZH2 and H3K27me3 in the PFC. (I) RT-qPCR analysis of *Tph2* mRNA expression. (J) Golgi staining analysis of synaptic structures. (K) TEM images showing synaptic ultrastructure. (L) FST, TST and SPT assessing depressive behaviors. * vs. FMT-Control, *p < 0.05, **p < 0.01; ^#^ vs. FMT-CUMS, *^#^p <* 0.05, *^##^p <* 0.01.

To further identify the specific microbes responsible for the decreased inosine levels in mice, we performed metabolic tracing analysis of inosine using MetOrigin 2.0. The results revealed that multiple bacterial families were involved in inosine metabolism, including *f_Bacillaceae*, *f_Paenibacillaceae*, *f_Staphylococcaceae*, *f_Streptococcaceae*, *f_Lachnospiraceae*, *f_Oscillospiraceae*, and *f_Erysipelotrichaceae* (Figure 6D). Additionally, 16S rRNA sequencing was conducted. PCA demonstrated distinct differences in gut microbiota composition between the control and CUMS mice (Figure 6A). Shannon indices indicated a reduction in gut microbial abundance in CUMS mice (Figure 6B). Further, Lefse analysis revealed that the relative abundance of *f_Erysipelotrichaceae*, *f_Oscillospiraceae*, and *f_Lachnospiraceae* was significantly reduced in CUMS mice (Figure 6C). Notably, these bacterial families are capable of catalyzing hypoxanthine into inosine *via* purine nucleoside phosphorylase (Figure 6D-E). Moreover, previous studies have shown that alterations in *f_Lachnospiraceae*, *f_Oscillospiraceae*, and *f_Erysipelotrichaceae* abundance are associated with neurological disorders 51-54. Therefore, we hypothesize that the decreased abundance of these bacteria may lead to reduced inosine levels, which in turn activates EZH2 transcription.

To validate the above hypothesis, we administered *R. hominis* to FMT-CUMS mice *via* oral gavage. *R. hominis* belongs to the *f_Lachnospiraceae* and is considered a beneficial probiotic 55. Importantly, UniProt confirms that *R. hominis* contains purine nucleoside phosphorylase, supporting its potential to convert hypoxanthine into inosine (Figure 6F and S6D). Consistent with our predictions, *R. hominis* supplementation increased inosine levels in the gut contents, serum and PFC of FMT-CUMS mice (Figure 6G), along with a decrease in EZH2 and H3K27me3 levels (Figure 6H). Furthermore, *R. hominis* restored the expression of *Tph2*, *Htr1d*, *Htr2a*, *Htr3a*, *Htr5*, and *Htr7*, partially rescuing dendritic spine density, postsynaptic membrane thickness, and synaptic cleft width (Figure 6I-K and S6C). Additionally, we observed that *R. hominis* supplementation alleviated neuronal apoptosis and depressive behaviors in FMT-CUMS mice (Figure 6L and S6A-B). Collectively, these findings further support the notion that gut microbiota contributes to depressive behaviors and neuronal apoptosis by reducing inosine synthesis, thereby activating EZH2-mediated H3K27me3 reprogramming.

### Screening of the natural product library identified CA as a degrader of EZH2, which ameliorates gut microbiota-induced depressive behaviors

**Figure 7:**
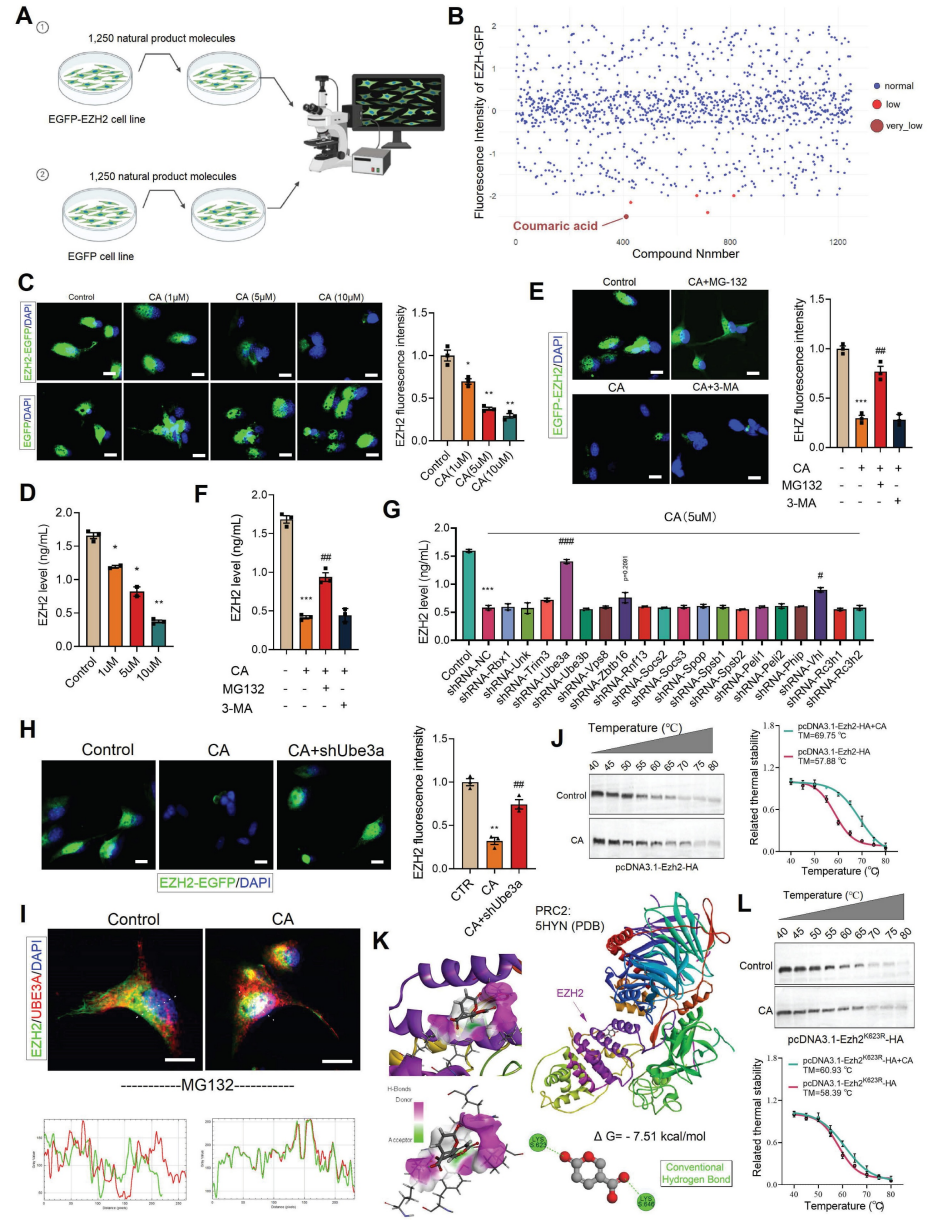
CA degrades EZH2 via UBE3A. (A) Flowchart of cell treatment with natural product library. (B) Cellular EZH-EGFP fluorescence intensity analysis. (C, E and H) Analysis of intracellular EZH2-GFP fluorescence levels. (D and F-G) ELISA quantification of EZH2 levels in cells. (I) Immunofluorescence colocalization analysis of EZH2 and UBE3A. (J and L) CETSA analysis demonstrating the interaction between CA and both EZH2 and EZH2^K623R^. (K) Molecular docking analysis illustrating the interaction mode of CA and EZH2. * vs. Control, *p < 0.05, **p < 0.01, ***p < 0.001; ^#^ vs. CA01, *^##^p <* 0.01, *^###^p <* 0.001.

We have demonstrated that EZH2 plays a critical role in gut-brain axis-related depression. Therefore, we sought to screen drug candidates that could inhibit EZH2 and alleviate depressive symptoms. To this end, we constructed a stable cell line expressing EZH2 fused with EGFP (EZH2-EGFP). Using a natural product library containing 1,250 compounds, we treated the cells for 48 hours (Figure 7A). Among the candidates, coumaric acid (CA) was identified as significantly reducing the fluorescence intensity of EZH2-EGFP cells (Figure 7B). Furthermore, we treated the cells stably expressing either EGFP-EZH2 or EGFP with varying concentrations of CA for 48 hours. The results demonstrated that CA effectively degraded EZH2 in a concentration-dependent manner (Figure 7C). Moreover, CA treatment did not affect EGFP protein levels, ruling out the possibility of false positives due to EZH2 mRNA downregulation or EGFP protein reduction (Figure 7C). Further quantitative analysis *via* ELISA showed that CA reduced EZH2 levels from 1.7 ng/mL to 0.4 ng/mL (Figure 7D).

Given the substantial differences between *in vitro* and *in vivo* environments, we further investigated the *in vivo* activity of CA. Mice were intraperitoneally injected with CA once daily for two weeks. Compared to the saline-injected group, CA-treated mice exhibited reduced EZH2 levels in the PFC (Figure S8A). Additionally, we assessed the toxicity of CA both *in vitro* and *in vivo*. HT22 cells were treated with CA for 12, 24, and 48 hours, and CCK8 assays indicated no cytotoxic effects (Figure S7A). *In vivo* toxicity was evaluated by analyzing the expression of key functional genes across multiple organs, including the liver (*Hmgcr, Fasn, Hnf4a, G6pase*), cerebral cortex (*Sox2, Syntaxin, Pcna, Igf1*), cartilage (*Runx2, Ocn, Col2al*), testicle (*Star, P450scc*), and skeletal muscle (*Pax7, Sox10*). The results indicated that CA did not affect the expression of these functional genes (Figure S7C). In addition, CA had no effect on the body weight of mice (Figure S7B). In conclusion, these findings demonstrate that CA effectively degrades EZH2 both* in vitro* and *in vivo* without inducing toxic effects.

To further investigate the potential therapeutic effects of CA *in vivo*, we administered intraperitoneal injections of CA (once daily) for two weeks to FMT-CUMS mice and assessed its impact on depressive-like behaviors. Behavioral analyses revealed that CA increased sucrose preference in FMT-CUMS mice and shortened immobility time in both the TST and FST (Figure S8B). At the molecular level, CA shortened synaptic cleft width and restored dendritic spine density and branching complexity (Figure S8C-E). Additionally, CA restored the expressions of *Tph2, Htr1d, Htr2a, Htr3a, Htr5,* and *Htr7*, and enhanced 5-HT synthesis (Figure S7D and S8F-G). Moreover, CA reduced the number of apoptotic neurons in FMT-CUMS mice (Figure S8H). Taken together, these findings demonstrate that CA effectively alleviates depressive-like behavior and neuronal damage, highlighting its potential as a therapeutic agent for depression.

### CA induces EZH2 degradation by promoting its interaction with UBE3A

Protein degradation primarily relies on two major pathways: the ubiquitin-proteasome system (UPS) and the autophagy-lysosome pathway. To determine the degradation mechanism of EZH2 induced by CA, we treated EZH2-EGFP cells with the proteasome inhibitor MG132 and the autophagy inhibitor 3-MA. MG132 significantly reversed the EZH2 degradation induced by CA, whereas 3-MA had no effect (Figure 7E). Furthermore, ELISA quantification of EZH2 levels showed that MG132 restored EZH2 expression from 0.4 ng/mL to 1.0 ng/mL, while 3-MA had no significant impact (Figure 7F). These results indicate that CA mediates EZH2 degradation *via* the UPS.

To further investigate the potential molecular mechanism by which CA induces EZH2 degradation, we designed shRNA sequences targeting 19 different E3 ubiquitin ligases and cloned them into the pLKO.1 vector. These constructions were then co-transfected with psPAX2 and pMD.2G into 293T cells for lentiviral packaging. Subsequently, each of these E3 ligases was individually knocked down in cells, followed by CA treatment for 48 hours. ELISA-based quantification revealed that *Ube3a* knockdown significantly reversed CA -induced EZH2 degradation (p < 0.001), while *Vhl* knockdown led to a slight reversal (p = 0.0256) (Figure 7G). Furthermore, in Ube3a-knockout cells stably expressing EZH2-EGFP, CA treatment failed to reduce EZH2-EGFP levels (Figure 7H), confirming that UBE3A mediates CA-induced EZH2 degradation.

We speculate that CA may degrade EZH2 by promoting its interaction with UBE3A. To validate this hypothesis, we first treated cells with MG132 to block EZH2 degradation. Immunofluorescence co-localization analysis revealed that CA promotes the interaction between EZH2 and UBE3A (Figure 7I). To further study the direct interaction between CA and EZH2, we performed cellular thermal shift assays (CETSA). The melting temperature (TM) of EZH2 was determined to be 57.88 °C under basal conditions, whereas CA treatment increased the TM to 69.75 °C, providing strong evidence of a direct interaction between CA and EZH2 (Figure 7J). To investigate this interaction, we utilized molecular docking and computational analyses, including Discovery Studio and PyMOL. Our results indicated that CA forms hydrogen bonds with residues K623 and K646 on EZH2 (Figure 7K). To validate these binding sites, we generated *in vitro* mutants EZH2^K623R^. CETSA demonstrated that the TM of mutant EZH2^K623R^ remained largely unchanged compared to EZH2^WT^, suggesting that these mutations do not significantly affect stability of EZH2. Next, cells were treated with CA and transfected with pcDNA3.1-EZH2^K623R^-HA, followed by protein extraction and CETSA. The results showed that CA slightly increased the TM of EZH2^K623R^, confirming that K623 is the direct binding site of CA on EZH2 (Figure 7L).

## Discussion

Gut microbiota and their metabolites play essential roles in neurodevelopment and functional regulation, and their dysbiosis has been closely linked to a variety of neuropsychiatric disorders, including depression 17-19. This study demonstrates the causal role of microbial dysbiosis in depressive behaviors: transplantation of fecal microbiota from CUMS-induced depressive mice to recipient mice induces significant depressive phenotypes. A key finding highlights the histone methyltransferase EZH2 as a critical mediator. EZH2 expression was markedly upregulated in the PFC of FMT-CUMS mice and positively correlated with neuronal apoptosis, synaptic injury, and behavioral impairments. Importantly, neuron-specific conditional knockout of EZH2 effectively reversed the pathological phenotypes induced by FMT, establishing EZH2 as a central player in mediating gut microbiota dysbiosis-induced neurodegeneration and depressive-like behavior.

As the core catalytic subunit of the PRC2 complex, EZH2 mediates H3K27me3 modification to silence downstream target genes 56, 57. Our results revealed a global reprogramming of H3K27me3 in the PFC of FMT-CUMS mice, with significant enrichment at gene loci involved in neurotransmitter signaling pathways, particularly serotonergic synapses and serotonin metabolism. Focusing on the serotonergic system—the foundation of the monoamine hypothesis of depression—we observed a significant reduction in 5-HT levels in the PFC following FMT, along with H3K27me3-mediated transcriptional repression of key genes including *Tph2, Htr1d, Htr2a, Htr3a, Htr6, and Htr7*. Among these, suppression of *Tph2*, the rate-limiting enzyme for 5-HT synthesis, was especially critical. Neuron-specific EZH2 deletion reduced H3K27me3 enrichment at the *Tph2* locus, restored its expression and 5-HT production, and rescued the associated neurobehavioral deficits. Conversely, forced expression of *Tph2* in FMT-CUMS mice equally effectively restored 5-HT levels and ameliorated depressive phenotypes. Collectively, these findings establish the EZH2/H3K27me3 axis as a molecular hub through which gut microbiota dysbiosis impairs Tph2 expression and 5-HT synthesis, driving depressive behaviors.

We further investigated the upstream mechanisms by which gut microbiota regulate EZH2 expression. Metabolomic profiling revealed a significant reduction in inosine levels in the gut, serum, and PFC of FMT-CUMS mice. Inosine has previously been reported to possess neuroprotective and antidepressant properties 58, 59. In this study, *in vitro* experiments confirmed that inosine directly inhibits EZH2 transcription. Further analysis showed that inosine deficiency weakened activation of its receptor A2aR and the downstream cAMP-PKA-CREB signaling pathway, which in turn further inhibited the transcriptional activation of EZH2. Mechanistically, inosine-A2aR signaling promotes CREB activation, which enhances its interaction with HDAC3 to catalyze H3K9ac deacetylation, thereby suppressing EZH2 transcriptional activity. These results delineate a signaling cascade: gut microbiota dysbiosis—reduced inosine—attenuated A2aR-PKA-CREB signaling—diminished CREB/HDAC3 complex formation—increased H3K9ac modification—EZH2 transcriptional activation.

Upstream microbial analysis identified specific bacterial families with hypoxanthine metabolic potential—*f_Lachnospiraceae*, *f_Oscillospiraceae* and *f_Erysipelotrichaceae*—as major producers of inosine. These taxa were significantly reduced in the FMT-CUMS mouse model. Functional validation was performed by oral supplementation of *R. hominis*, a representative species of *f_Lachnospiraceae* 55. Administration of *R. hominis* effectively restored inosine levels, reduced EZH2 expression, and significantly improved neuronal structure and behavioral phenotypes in FMT-CUMS mice. These findings establish that loss of specific functional microbiota drives inosine depletion and subsequent EZH2-mediated depression pathology.

Given EZH2's central role in this pathway, it represents a promising therapeutic target. However, current EZH2 inhibitors (such as Tazverik and Valemetostat) are primarily developed as chemotherapeutic agents and exhibit considerable cytotoxicity. Natural product-based compound libraries provide an efficient strategy for identifying novel agonists, inhibitors, or degraders targeting specific proteins 60, 61. Here, screening of a natural compound library led to the identification of a novel EZH2 degrader, CA. CA exhibited low toxicity and effectively induced EZH2 degradation, significantly improving depressive behaviors and reducing neuronal apoptosis in vivo. Mechanistically, CA promotes EZH2 degradation via the UPS in a UBE3A-dependent manner: CA directly binds EZH2 at K623 and K646 residues, facilitating its spatial association with the E3 ligase UBE3A. This discovery provides proof-of-concept and a lead compound for the development of low-toxicity, degradation-based antidepressant therapies.

In summary, this study reveals a novel gut microbiota-brain pathway underlying depression: the depletion of specific microbial taxa (*f_Lachnospiraceae, f_Oscillospiraceae and f_Erysipelotrichaceae*) leads to reduced inosine levels, attenuating A2aR-PKA-CREB-HDAC3 signaling, increasing H3K9ac, and promoting EZH2 transcription. Subsequently, EZH2 induces H3K27me3 reprogramming, repressing genes critical for serotonergic synapse function, and ultimately causing depressive behavior. These findings provide mechanistic insights into depression at the molecular level. Moreover, through natural product screening, we propose a therapeutic strategy involving the targeted degradation of EZH2 using CA. This approach not only elucidates the precise mechanism underlying the antidepressant effect of CA but also supports its potential as a candidate antidepressant. Nevertheless, several questions remain to be addressed in future research: although we demonstrated that gut microbiota mediates inosine depletion, it remains unclear whether endogenous inosine biosynthesis is altered or contributes to this process. Neuronal-specific EZH2 knockout exhibited only a weak trend toward improvement in gut microbiota—mediated anxiety-like behaviors, suggesting a potential, albeit limited, link between EZH2 and anxiety phenotypes. Additionally, whether inosine levels and associated microbial changes are consistent in human patients with depression also warrants further investigation.

## Supplementary Material

Supplementary methods, figures and tables.

## Abbreviations

EZH2: enhancer of zeste homolog 2
H3K27me3: trimethylation of lysine 27 on histone H3
CA: coumaric acid
A2aR: adenosine A2a receptor
CREB: cAMP responsive element binding protein 1
HDAC3: histone deacetylase 3
MDD: major depressive disorder
FMT: fecal microbiota transplantation
UBE3A: ubiquitin protein ligase E3A
CUMS: chronic unpredictable mild stress
