# Supplementary material for: EZH2-mediated H3K27me3 links microbial inosine loss to depression: a gut-brain epigenetic switch
Source: Theranostics. 2025 Sep 12;15(18):9969–86. doi: 10.7150/thno.120824 (PMC12486992; doi:10.7150/thno.120824)
Supplement: Supplementary file 1 — Supplementary methods, figures and tables. [file thnov15p9969s1.pdf]

Supplementary materials

EZH2-mediated H3K27me3 links microbial inosine loss to depression: a gut-brain epigenetic switch

Sen Zhu [1 #], Xuan Li [2 #], Ying Yu [3], Xiaoyi Han [1], Fang Yang [1], Mengxi Lu [1], Gaole Dai [1], Liang Guo [2 *], Dan Xu [1,4,5 *]

[1] Department of Obstetrics, Zhongnan Hospital of Wuhan University, School of Pharmaceutical Sciences, Wuhan University, Wuhan, 430071, China.

[2] Department of Plastic Surgery, Zhongnan Hospital of Wuhan University, Wuhan University, Wuhan, 430071, China.

[3] Department of Neurology, Renmin Hospital of Wuhan University, Wuhan, 430060, China.

[4] Hubei Provincial Key Laboratory of Developmentally Originated Disease, Wuhan, 430071, China.

[5] Ministry of Education Key Laboratory of Combinatorial Biosynthesis and Drug Discovery, Wuhan, 430071, China.

* Corresponding author: Dan Xu (xuyidan70188@whu.edu.cn).

[#] These authors contribute equally.

Supplementary figure 1

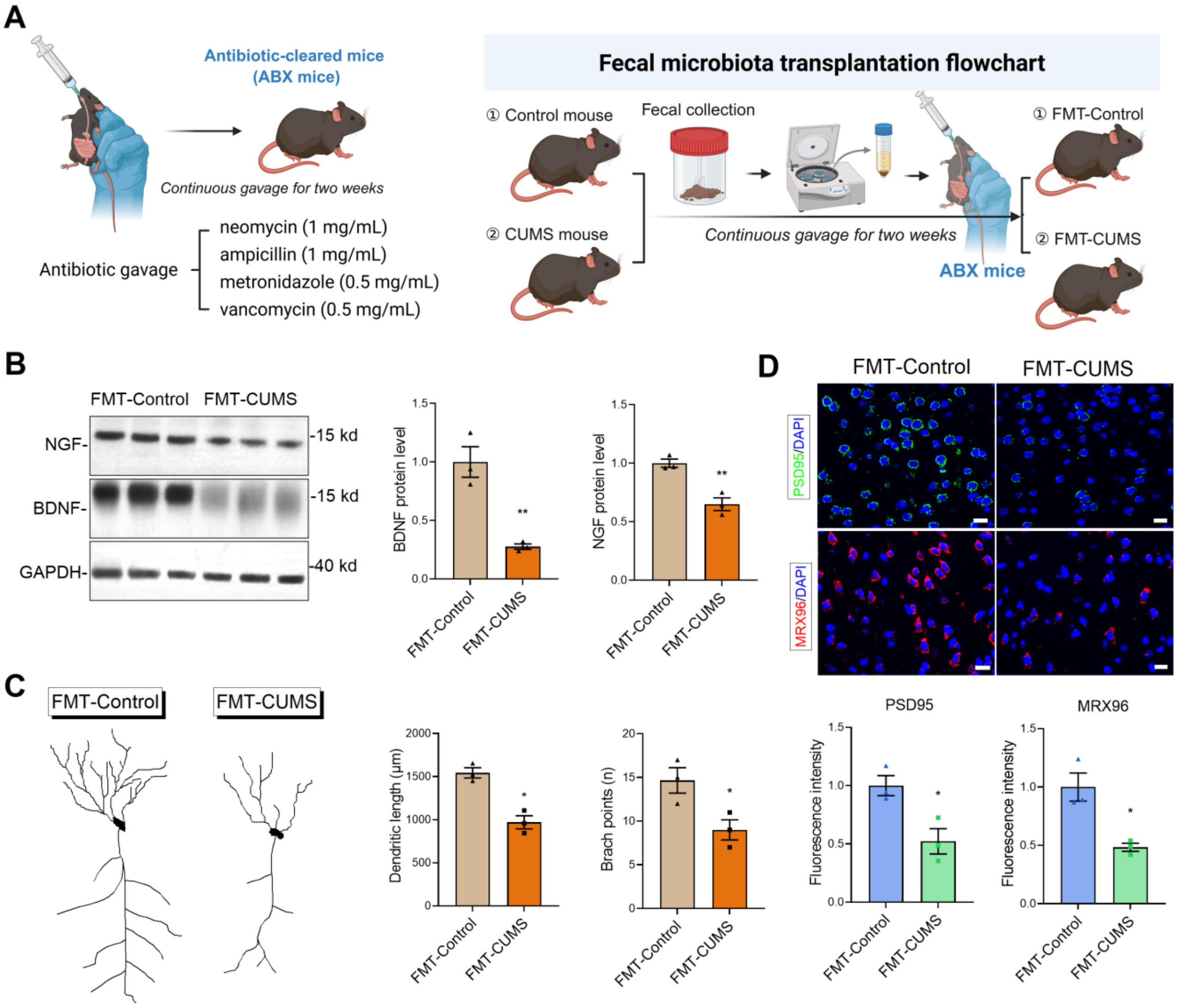

Supplementary figure 1. Alterations in synaptic-related indicators in the PFC of FMT-CUMS mice. (A) Fecal microbiota transplantation flowchart. (B) Western blot analysis of NGF and BDNF levels in PFC. (C) Representative image of Golgi staining reconstruction. Statistics on dendrite length and number of branches. (D) Analysis of intracellular PSD95 and MRX96 fluorescence levels. * *vs.* FMT-Control, *$p<0.05$, **$p<0.001$.

Supplementary figure 2

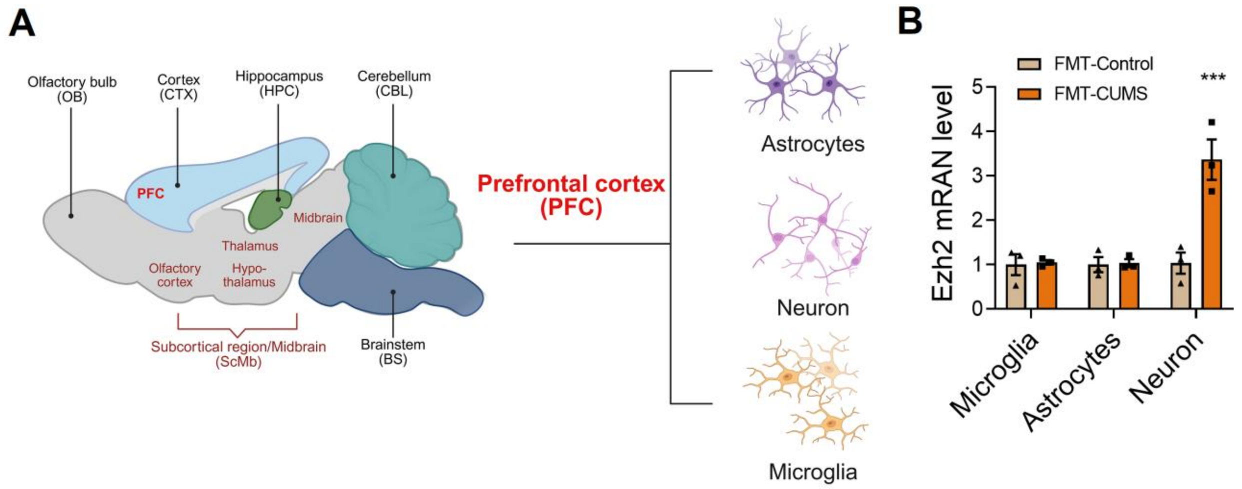

Supplementary figure 2. Expression of Ezh2 in the PFC of FMT-CUMS mice. (A) Microglia, astrocytes, and neurons are isolated from the PFC region. (B) RT-qPCR analysis of *Ezh2* mRNA expression levels. * *vs*. FMT-Control, ***p<0.001.

Supplementary figure 3

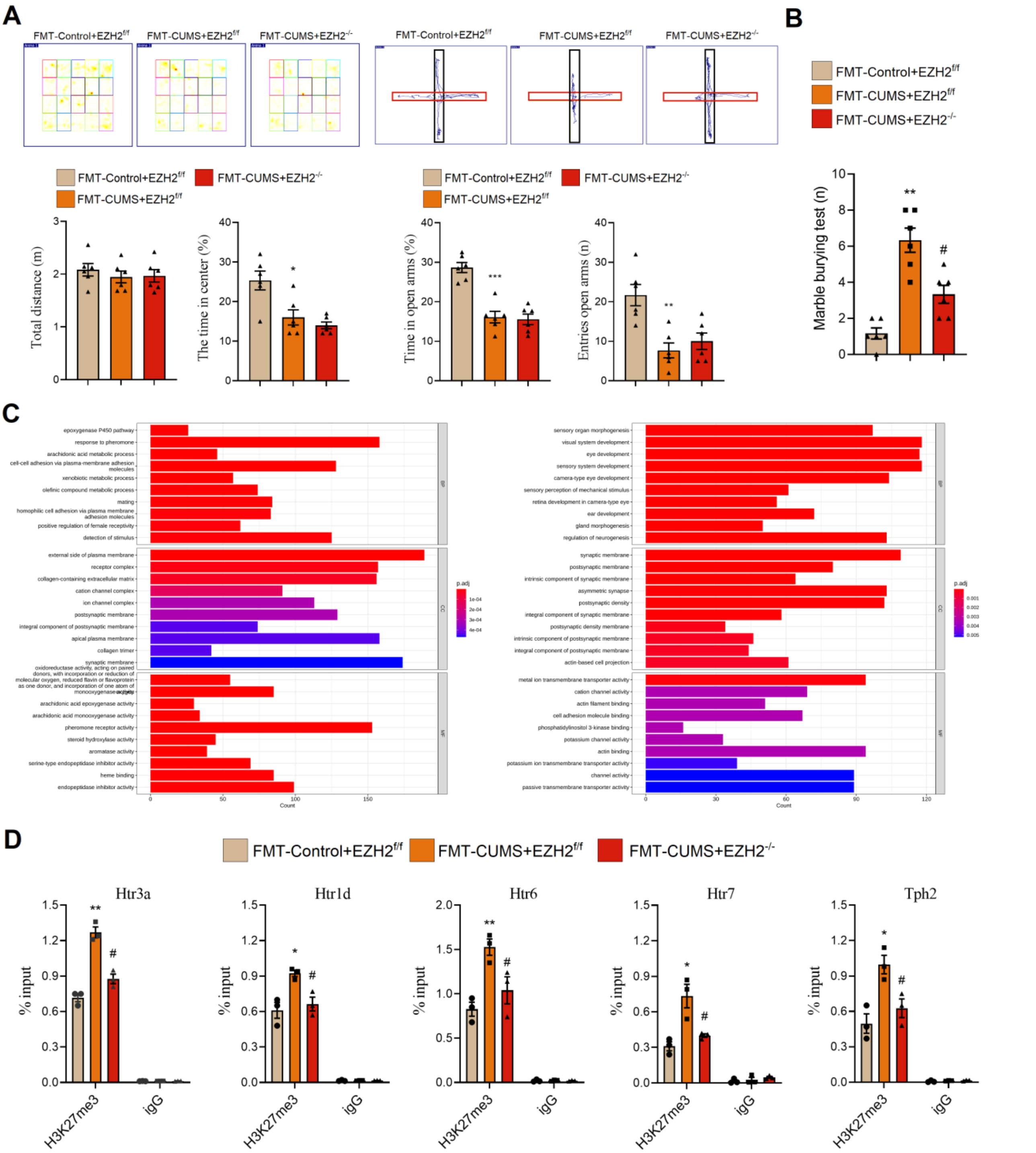

Supplementary figure 3. Effect of neuronal EZH2 knockout on H3K27me3 levels. (A) OFT and EPMT. (B) Marble burying test. Number of marbles buried by mice. (C) GO analysis of differential H3K27me3-modified genes. (D) CUT&Tag-qPCR was used to analyze H3K27me3 modification levels at *Htr3a, Htr1d, Htr6, Htr7,* and *Htr2a* loci. * *vs.* FMT-Control+EZH2$^{f/f}$, *p<0.05, **p<0.01; $^{\#}$ *vs.* FMT-CUMS+EZH2$^{f/f}$, $^{\#}p$<0.05.

Supplementary figure 4

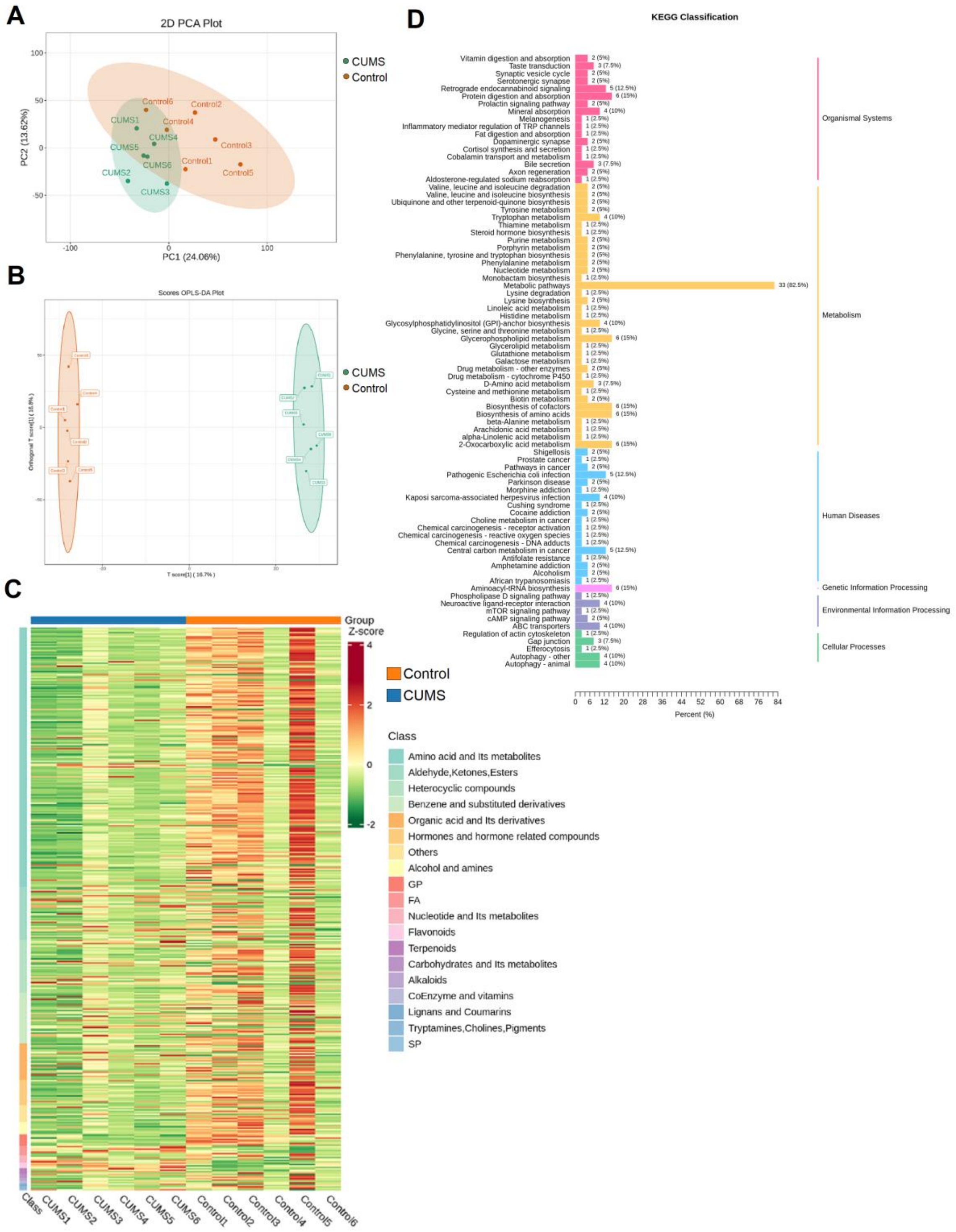

Supplementary figure 4. Untargeted metabolomics analysis of gut contents. (A) PCA illustrates the differences in gut microbial community structure between groups. (B) OPLS-DA plot demonstrates distinct metabolic profiles between groups. (C) Heatmap of differential metabolites. (D) KEGG enrichment analysis of differential metabolites.

Supplementary figure 5

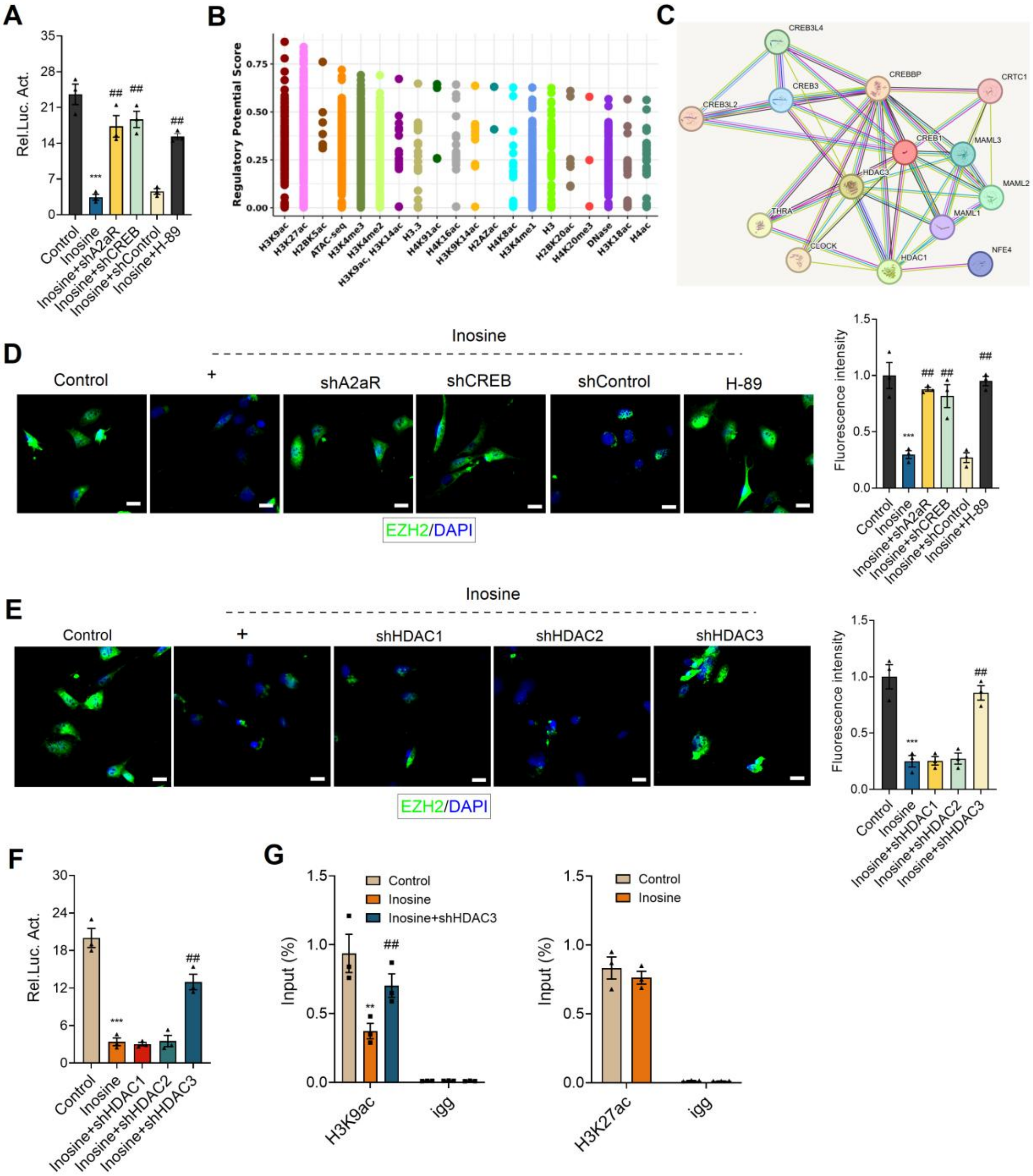

Supplementary figure 5. Inosine mediates the transcriptional inhibition of Ezh2 *via* the A2aR-cAMP-PKA-CREB-HDAC3 pathway. (A and F) Luciferase reporter assay was used to analyze the transcriptional activation of Ezh2. (B) Cistrome BD displays the epigenetic modification forms of *Ezh2*. (C) PPI reveals proteins interacting with CREB. (D-E) Analysis of intracellular EZH2 fluorescence levels. (G) ChIP-qPCR is used to analyze the level of H3K27ac at the *Ezh2* promoter region. * *vs.* Control, *p<0.05, **p<0.01; # *vs.* Inosine, #p<0.05, ##p<0.01.

Supplementary figure 6

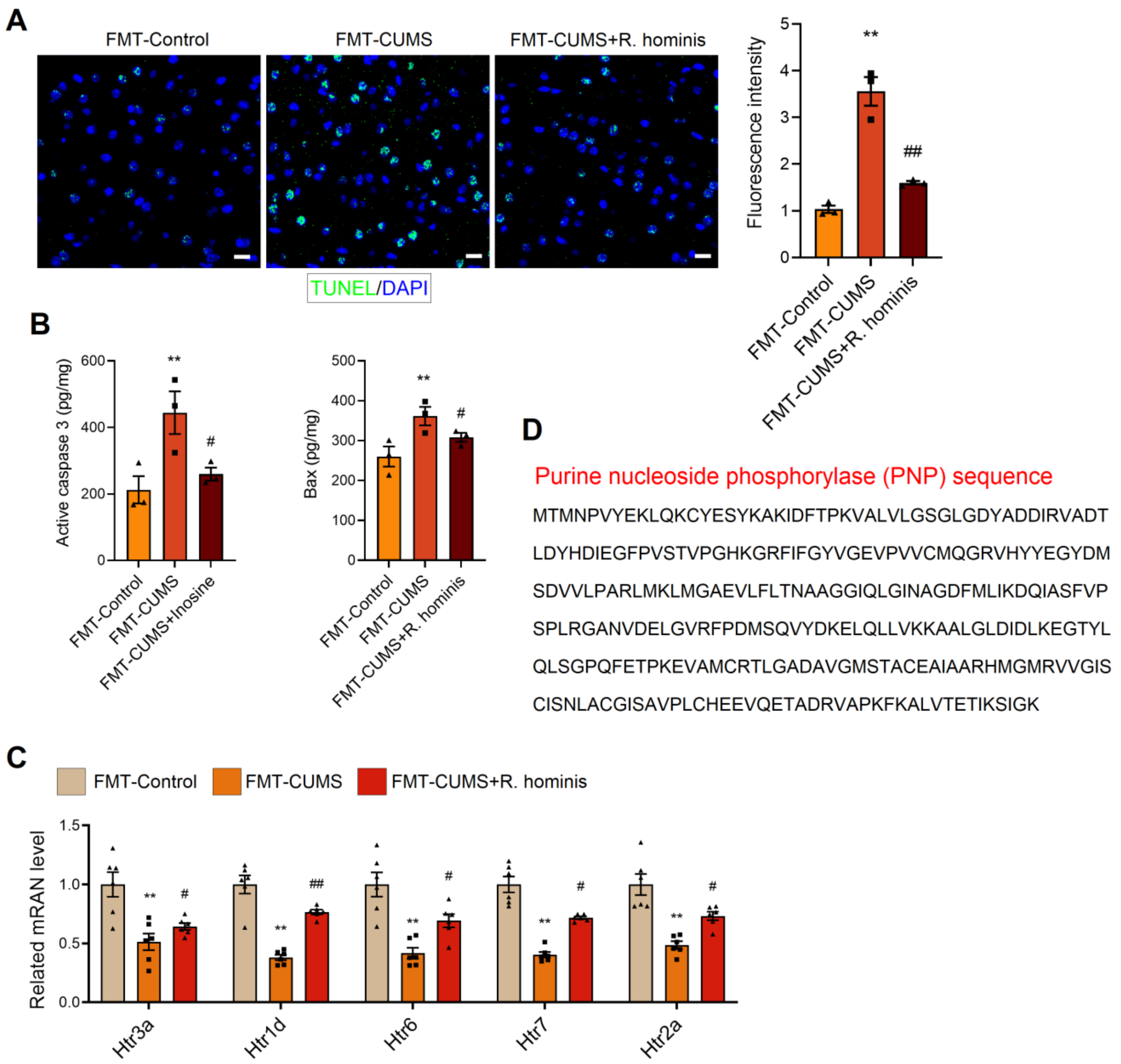

Supplementary figure 6. The effect of *R. hominis* on neuronal apoptosis in FMT-CUMS mice. (A) TUNEL staining for apoptotic cell detection. (B) ELISA analysis of activated caspase 3 and Bax protein levels. (C) RT-qPCR analysis of *Htr3a, Htr1d, Htr6, Htr7,* and *Htr2a* mRNA expression. (D) Amino acid sequence of PNP. * *vs.* FMT-Control, **p<0.01; $^{\#}$ *vs.* FMT-CUMS, $^{\#}p$<0.05, $^{\#\#}p$<0.01.

Supplementary figure 7

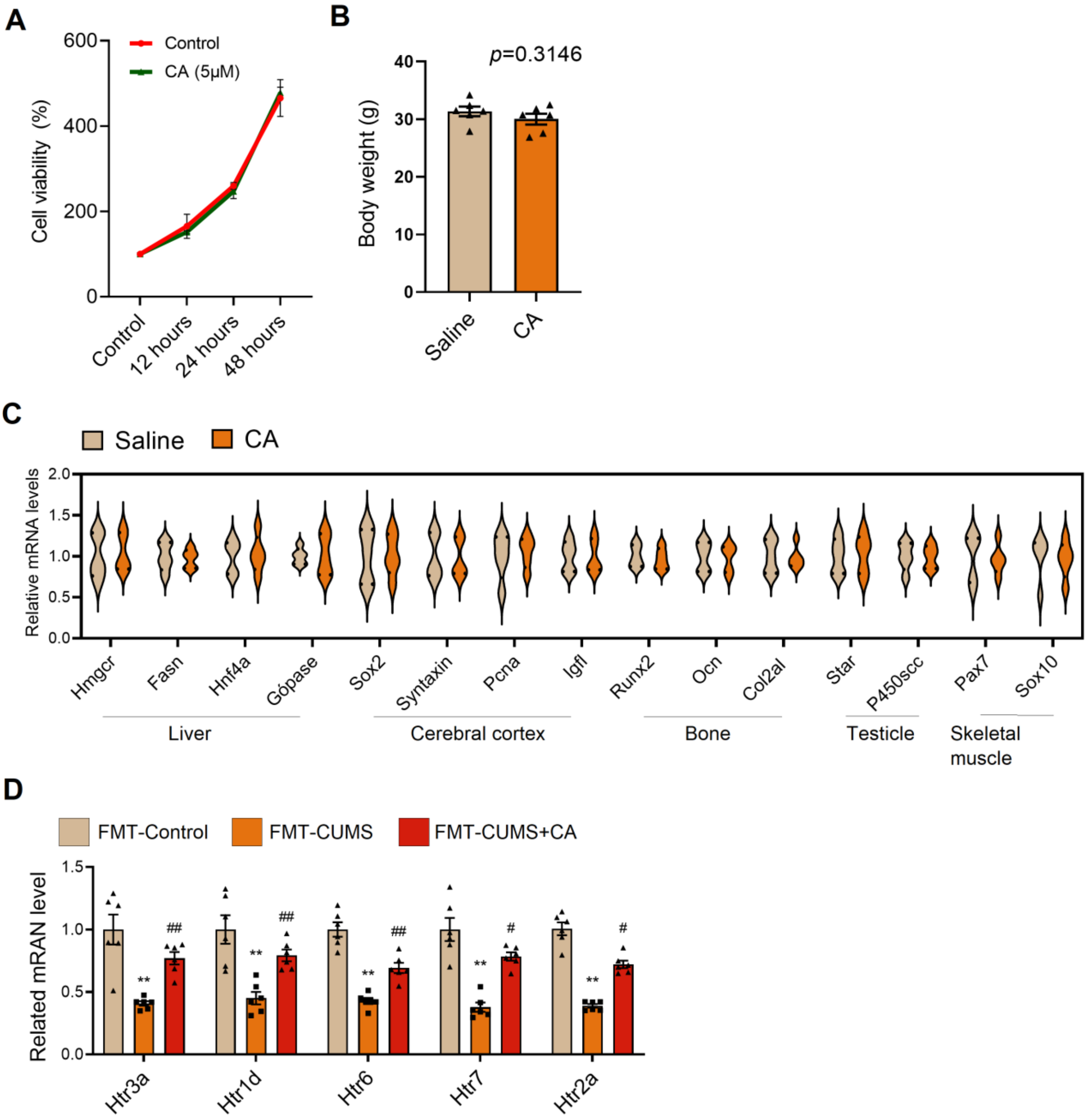

Supplementary figure 7. *In vivo* and *in vitro* toxicity testing of CA. (A) CCK8 analysis of the toxic effects of CA on cells. (B) The effect of CA on mouse body weight. (C) RT-qPCR analysis of relevant functional genes in liver, cortical brain, bone, testis, and muscle. (D) RT-qPCR analysis of *Htr3a, Htr1d, Htr6, Htr7,* and *Htr2a* mRNA expression. * *vs.* FMT-Control, *p<0.05; **p<0.01; # *vs.* FMT-CUMS, #p<0.05, ##p<0.01.

Supplementary figure 8

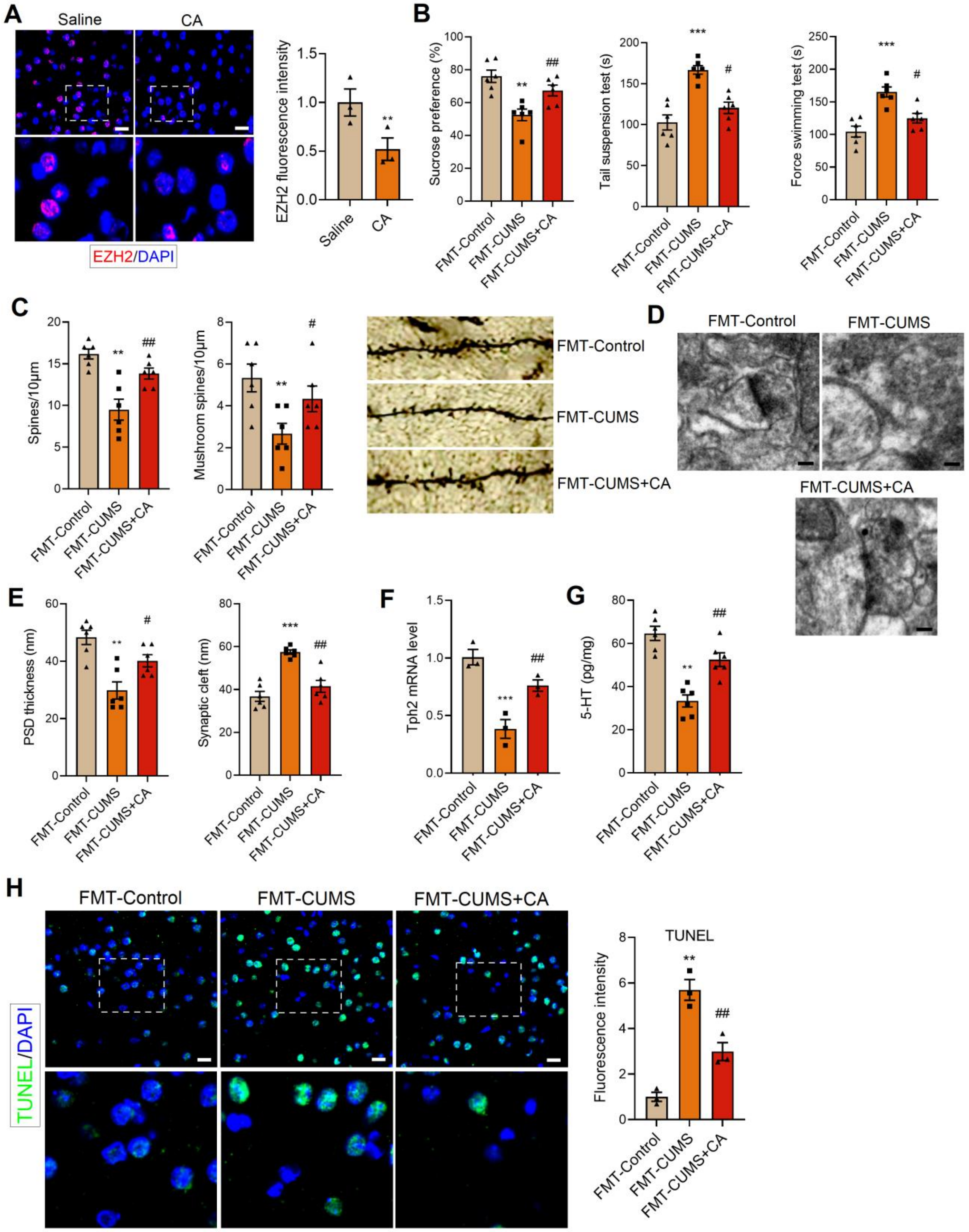

Supplementary figure 8. CA improves depressive-like behaviors and neuronal apoptosis in FMT-CUMS mice. (A) Immunofluorescence staining of EZH2 in the PFC. (B) SPT, TST, and FST assessing

depressive behaviors. (C) Golgi staining analysis of synaptic structures. (D-E) TEM images of synaptic ultrastructure. (F) RT-qPCR analysis of Tph2 mRNA levels. (G) ELISA quantification of 5-HT levels in the PFC. (H) TUNEL staining for neuronal apoptosis assessment. * *vs.* FMT-Control/Saline, *$p<0.05$, **$p<0.01$; $^{\#}$ *vs.* FMT-CUMS, $^{\#}p<0.05$, $^{\#\#}p<0.01$.

Table S1: The list of metabolites.

Name	Manufacturer	Product code
Inosine	MedChemExpress	HY-N0092
3-MA	MedChemExpress	HY-19312
MG132	Sellect	S2619
CA	MedChemExpress	HY-32004
Ampicillin	Beyotime	ST007
Neomycin	Beyotime	ST2528
Metronidazole	MedChemExpress	HY-B0318
Vancomycin	MedChemExpress	HY-B0671

Table S2: The list of antibodies for Western blotting and Immunofluorescence.

Antibody	Manufacturer	Product code
H3K27me3	Abclonal	A16846
EZH2	CST	#5246
TPH2	CST	#51124
HA	CST	#3724

Table S3: The sequence of primer for RT-qPCR.

Target	Forward Primer (5'–3')	Reverse Primer (5'–3')
Tph2	*GCAAGACAGCGGTAGTGTTCT*	*CAGTCCACGAAGATTTCGACTT*
Htr3a	*TCCTGAGGACTTCGACAATGT*	*CCCCACGTCCACAAACTCAT*
Htr1d	*TCCAAACCAGTCCCTAGAAGG*	*CACCACGAGCGAGATTCTGA*
Htr6	*CCAGCCTGCCTTATGTCCTC*	*CTGGGCACCTGCAAGGTTT*
Htr7	*TGCGGGGAGCAGATCAACTA*	*GACAAAGCACACCGAGATCAC*
Htr2a	*TAATGCAATTAGGTGACGACTCG*	*GAGGCTTCGGAAGTGTTAGCA*

Hmgcr	*TGTTCACCGGCAACAACAAGA*	*CCGCGTTATCGTCAGGATGA*
Fasn	*GGAGGTGGTGATAGCCGGTAT*	*TGGGTAATCCATAGAGCCCAG*
Hnf4a	*ATGCGACTCTCTAAAACCCTTG*	*ACCTTCAGATGGGGACGTGT*
Sox2	*AGCAATGGTTCTTATGTTGGACG*	*TGCTTTGTCCGTATCCAGTGC*
Syntaxin	*GACCCCTGGTTCTCCACATAC*	*AGCAGGTTCTGTCTTCGATCC*
Pcna	*TTTGAGGCACGCCTGATCC*	*GGAGACGTGAGACGAGTCCAT*
Runx2	*GACTGTGGTTACCGTCATGGC*	*ACTTGGTTTTTCATAACAGCGGA*
Col2al	*GGGTCACAGAGGTTACCCAG*	*ACCAGGGGAACCACTCTCAC*
Star	*TGCCCATCATTTCATTCATCCTT*	*AAAAGCGGTTTCTCACTCTCC*
P450scc	*AGGTCCTTCAATGAGATCCCTT*	*TCCCTGTAAATGGGGCCATAC*
Pax7	*TGGGGTCTTCATCAACGGTC*	*ATCGGCACAGAATCTTGGAGA*
Sox10	*CGGACGATGACAAGTTCCCC*	*GTGAGGGTACTGGTCGGCT*

Table S4: The sequence of shRNA.

Target	shRNA sequence
A2aR	*CGAAGGGCATCATTGCGATTT*
Creb	*GCCTGAAAGCAACTACAGAAT*
Hdac1	*GCTTGGGTAATAGCAGCCATT*
Hdac2	*CCCAATGAGTTGCCATATAAT*
Hdac3	*CGTGGCTCTCTGAAACCTTAA*
Ube3a	*CGGAGAATGATGGAAACATTT*
Rbx1	*CCTGGGACATTGTGGTTGATA*
Unk	*GCAGTCAGTGAAATGCCTTAA*
Trim3	*CCCACGGTTGCACTTTATTTA*

Ube3b	*CGTCCTACATTCACGAGAATT*
Vps8	*CCAGAGAAATTCACAGAATTT*
Zbtb16	*CGTGGTCTCCTGCCGTTAATA*
Rnf1	*GCCTGGAAGGTGTCAGCGAAA*
Socs2	*CCTACTAACTATATCCGTTAA*
Socs3	*TCTTCACGTTGAGCGTCAAGA*
Spop	*CGCTTGAAGCAATCCTAAGAT*
Spsb1	*GTTGGGTACACAACCCTTGTA*
Spsb2	*GCTGGCCTAGACAACTTTAAA*
Peli1	*GCTCCTTTGAATATGCAATTT*
Peli2	*GCTCCTACTCAGAAGCACATA*
Phip	*TTGAATGTAGGTCGCTAATTT*
Vhl	*GTTAACCAAACGGAGCTGTTT*
Rc3h1	*CGCACAGTTACAGAGCTCATT*
Rc3h2	*CAACTCATTAGATGGATATTA*

Supplement materials and methods

Behavioral tests

Forced swim test (FST): Mice were placed in a glass cylinder (25 cm high, 10 cm diameter) filled with water to a depth of 20 cm, maintained at 24 ± 1°C. Mice were immersed in the water for 6 minutes, and the duration of immobility during the last 4 minutes was recorded. Immobility was defined as floating or making minimal movements to keep the head above water.

Tail suspension test (TST): Mice were gently removed from their cages, and their tails were quickly secured with medical tape 1 cm from the tail tip to minimize stress. The tail was then suspended from a hanging rod, with the tip of the tail approximately 30 cm above the ground, causing the mouse to hang in an inverted position. Mice were suspended for 6 minutes, and the duration of immobility during the last 4 minutes was recorded. Immobility was defined as the mouse stopping struggling and maintaining a vertically inverted position.

Sucrose preference experiment (SPT): mice underwent 48-hour sucrose training, beginning with dual 1% sucrose bottles for 24 hours, followed by one sucrose bottle and one water bottle with position rotation every 12 hours. After 24-hour food and water restriction, sucrose preference was assessed by measuring 24-hour consumption from two bottles (1% sucrose vs. water), with positions alternated at 12-hour intervals to control for side preference.

Marble burying test (MBT): Mice were placed in a 40 cm × 20 cm arena with a bedding depth of 3 cm, containing 20 glass marbles arranged in a 4 × 5 grid. After 20 minutes, the mice were gently removed, and the number of marbles buried was recorded. A marble was considered buried if more than 50% of its surface area was covered by bedding.

Open field test (OFT): the test mice were placed in an opaque acrylic box with an open top (50 × 50 × 40 cm) and allowed to freely explore the environment. The movement trajectories of each mouse were recorded over a 5-minute session, with the apparatus cleaned using 75% ethanol between tests to prevent olfactory interference. Finally, analysis was performed using the Smart v3.0 video tracking system (Panlab, Spain).

Elevated plus maze test (EMPT): the EPM featured two open and two enclosed arms (35 × 5 × 15 cm^3) extending from a central platform. After ethanol (75%) cleaning to eliminate odor cues, mice were

positioned on the platform for a 5-minute exploration. Behavioral parameters, including arm preference and movement patterns, were quantified using the Smart v3.0 system.

Transmission electron microscopy (TEM)

The hippocampal tissue was fixed in electron microscope fixative, rinsed three times with 0.1 M phosphate buffer (15 min each), and post-fixed with 1% osmium acid for 2 hours at room temperature. After three more rinses, the tissue underwent graded alcohol dehydration (20 min each) followed by two rounds of 100% acetone dehydration (15 min each). It was then embedded, polymerized, ultrathin-sectioned (60–80 nm, Leica UC7, USA), and mounted on 150-mesh cuprum grids with formvar film. Staining involved 2% uranyl acetate (8 min, protected from light), three rinses in 70% ethanol and ddH_2O, followed by 2.6% lead citrate (8 min, CO_2-avoided) with three ddH_2O rinses. After drying overnight, imaging was performed using an HT7800 (HITACHI, Japan).

Microglia isolation and purification

Mice were euthanized, and the PFC tissues were dissected from the brain (*1*). The tissues were minced and digested with 0.125% EDTA-trypsin (Gibco, USA) at 37 °C for 20 min. Following digestion, the reaction was terminated, and the cell suspension was collected and centrifuged. The supernatant was discarded, and the pellet was resuspended in high-glucose DMEM supplemented with 10% fetal bovine serum (FBS) and 1% penicillin–streptomycin. The suspension was then filtered and seeded into T75 culture flasks at a density of 1×10^6 cells/mL. Cells were maintained at 37 °C in a humidified incubator with 5% CO_2, and the medium was replaced every 3 days. After one week, the flasks were placed on a constant-temperature shaker at 37 °C and agitated at 200 rpm for 2 hours. The supernatant containing detached microglia was collected, and the purity of microglia was verified by immunofluorescence staining for IBA1 and CX3CR1.

Neuron isolation and purification

PFC tissues were minced and digested with papain for 30 min, and the reaction was terminated with FBS. The suspension was sequentially filtered, centrifuged, and resuspended. Neurons were seeded at a density of 1×10^6 cells/mL into six-well plates pre-coated with poly-L-lysine (0.1 mg/mL, P6407, Thermo Fisher Scientific) and maintained at 37 °C in a humidified incubator with 5% CO_2. After 6 hours, the initial culture medium was replaced with serum-free Neurobasal medium supplemented with 2%

B27. Neuronal growth was monitored and recorded under a microscope. After 10 days of culture, purified neurons were obtained, and their purity was verified by immunofluorescence staining for PSD95 and MAP2.

Astrocyte isolation and purification

PFC tissues were minced and digested with 0.25% hyaluronidase. The resulting suspension was centrifuged, resuspended, and passed through a 200-mesh cell strainer. The filtrate was centrifuged again, resuspended, and astrocytes were seeded into culture flasks at a density of approximately 1×10^6 cells/mL. Cells were maintained in DMEM/F12 medium supplemented with 10% FBS, 100 U/mL penicillin, and 50 μg/mL streptomycin, in a humidified incubator at 37 °C with 5% CO_2. Once the cells reached confluence, purification was performed by shaking the flasks at 260 rpm for 18 hours at 37 °C. The purity of astrocytes was verified by immunofluorescence staining for GFAP and ALDH1L1.

Immunofluorescence

The immunofluorescence procedure followed established methods. Briefly, paraffin-embedded mouse brain sections (5 μm) were dewaxed, rehydrated, and subjected to antigen retrieval. After blocking with BSA (Beyotime, China) for 30 min, sections were incubated overnight at 4°C with primary antibodies, then washed with PBS (3×5 min). Secondary antibodies were applied and incubated at room temperature for 50 min.

For cellular immunofluorescence, cells were fixed with 4% PFA for 15 min, permeabilized with 0.5% Triton X-100 (Beyotime, China) for 15 min at 24°C, blocked with goat serum, and incubated overnight with primary antibodies. After a 1-hour incubation with fluorescent secondary antibodies, imaging was performed using a fluorescence microscope. Antibody details are in Table S2.

Enzyme-linked immunosorbent assay (ELISA)

Cortical tissues from mice were dissected, homogenized, and centrifuged to collect the supernatant. ELISA was performed to measure 5-HT, NGF, and BDNF levels according to the manufacturer's instructions. Mouse NGF and BDNF ELISA kits were purchased from Jin'ning Biological Co., Ltd. (Shanghai, China).

Apoptosis analysis

Brain tissues were sectioned into 5 μm slices and stored at 4°C. Cortical apoptosis was assessed using a TUNEL staining apoptosis detection kit (Beyotime, China) following the manufacturer's instructions. Briefly, after deparaffinization and rehydration, sections were washed three times with 0.1 M PBS and treated with Proteinase K (Beyotime, China) at 37°C for 15 minutes. After PBS washing, the sections were incubated with the TUNEL reaction mixture in a dark, humidified chamber at 37°C for 2 hours, followed by DAPI counterstaining of nuclei. Finally, fluorescence microscopy was used for imaging.

Metabolomics analysis

A tissue sample (10 mg), milled with liquid nitrogen, was placed into EP tube. Subsequently, 100 μL of 80% methanol was added to the tube, after vortexing. The sample was then incubated on an ice bath for 5 min, followed by centrifugation at 15,000 g for 20 min at 4°C. A specific volume of the resulting supernatant was appropriately diluted with mass spectrometry-grade water until the methanol content reached 53%. Additional centrifugation at 15,000 g for 20 minutes at 4°C was conducted to collect the supernatant, which was subsequently injected into the sample for LC-MS. Using the KEGG database (https://www.genome.jp/kegg/pathway.html), the HMDB database (https://hmdb.ca/metabolites), and the LIPID Maps database (http://www.lipidmaps.org/) facilitated the annotation of the identified metabolites. Metabolomic testing and analysis were conducted at Maiwei Inc (Wuhan China).

Gut microbiota profiling

The V3-V4 region of the bacteria's 16S rRNA gene was amplified by PCR with barcode-indexed primers (338F and 806R). Amplicons were then purified by gel extraction and quantified using QuantiFluor-ST. The purified amplicons were pooled in equimolar concentrations, and paired-end sequencing was performed using an Illumina MiSeq instrument (Illumina, USA). The raw sequencing data were separated into individual samples based on barcodes. Subsequently, the data was assembled using the FLASH software (version 250.1.2). The assembled tags underwent quality control to obtain clean tags, which were further filtered to eliminate chimeric sequences, resulting in a set of effective tags suitable for subsequent analysis. Regarding taxonomic annotation, the effective tags from all samples were clustered into operational taxonomic units (OTUs) using a 97% identity threshold by QIIME (version 1.8.0). The sequences of the OTUs were then annotated to identify different taxa by Mothur (version v.1.30). The species annotation results were visualized using KRONA for visualization. The community

structure of gut microbiota was assessed by α and β diversity. The Shannon index and Simpson index were used to evaluate α-diversity. Wilcoxon's rank-sum tests were used to determine the significance of differences in α diversity between groups. The β diversity was calculated based on the Bray-Curtis distances and displayed as principal coordinate analysis (PCoA) plots. Permutational multivariate analysis of variance (PERMANOVA) was conducted to determine the significance of differences in β diversity between groups. The R value refers to the extent to which a variable can explain the β diversity of the microbial communities. LEfSe was carried out to identify discriminating bacterial taxa between groups. The tests listed above were conducted on R (Version 3.6.3) or on the cloud platform of Maiwei Biology Information Technology Co., Ltd. (https://cloud.metware.cn/#/user/login).

RT-qPCR analysis

Total RNA was extracted from mouse hippocampal tissues using TRIzol reagent (Takara, Japan) and reverse-transcribed into cDNA with HiScript II Select qRT SuperMix (Vazyme, China) following the manufacturer's instructions. RT-qPCR was performed using SYBR premix (Vazyme, China) to quantify mRNA levels, analyzed by the $2^{\wedge-\Delta\Delta Ct}$ method. Primer sequences were obtained from Primebank, synthesized by Tianyi Biologicals (Wuhan, China). The sequences are referenced to table S3.

Western blot assay

PFC tissues were sheared and incubated with 200 μL of lysis solution for 40 min. Following centrifugation at 13,500 rpm for 7 min at 4°C, proteins were collected. The 160 μL protein was mixed with 40 μL loading buffer (Servicebio, China) and heated at 100 °C for 15 min. Subsequently, samples were either used for SDS-PAGE or stored at -80 °C. Proteins were supplemented with 1% PSFM (Servicebio, China) to prevent degradation. SDS-PAGE was conducted under specific conditions: a concentrated gel stage at 80V for 30 minutes and a separated gel stage at 120V for 60-90 minutes. Proteins were subsequently transferred from the gel to a PVDF membrane, which was briefly activated with methanol prior to the transfer. The transmembrane process was conducted at 300 mA, with varying transmembrane times (40-90 min) based on the target protein. Next, the proteins were blocked using a rapid blocking solution and incubated at 4°C with primary antibodies. The following day, the PVDF membrane was washed 3 times with TBST and then incubated with the HRP-labeled secondary antibody for 1 hour at room temperature, in succession to an additional three washes with TBST. Finally, a

chemiluminescent solution was added for signal development. The list of antibodies used is provided in Table S2.

Cell culture

HT22 and HeLa cells were cultured in DMEM high glucose medium supplemented with 10% FBS, 100 U/ml penicillin, and 100 mg/ml streptomycin at 37°C in air containing 5% CO2. When the cell confluence reached 85%–90%, the cells were digested with trypsin, collected by centrifugation, washed three times with PBS, resuspended in complete medium. Subsequently, the resuspended cells were then divided into two parts for further culture or seeded into well plates for subsequent experiments.

Cell line authentication and contamination testing

HT22 (mouse hippocampal neuron-derived) and HeLa (human cervical carcinoma) cell lines were purchased from Cyagen Biosciences Inc. in June 2023. Both cell lines were authenticated using short tandem repeat (STR) profiling, which confirmed a 100% match with the reference profiles in established cell line databases. The cells were routinely tested for mycoplasma contamination using the PCR-based MycoAlert™ Mycoplasma Detection Kit (Lonza), and all samples used in the experiments were confirmed to be negative. Research Resource Identifiers (RRIDs) are as follows: HT22 cells (RRID: CVCL_0321); HeLa cells (RRID: CVCL_0030).

Cell treatment and drug screening

Construction of EZH2-EGFP stable expression cells using HeLa cell line. Digest the EGFP or EGFP-EZH2 cells and seed them into 96-well or 12-well plates, ensuring the cell density reaches 60% the next day. Then, add CA dissolved in DMSO to the culture medium and continue incubating for 48 hours. Fluorescence intensity is detected using a fluorescence microscope or fluorescence plate reader, or proteins are extracted for CETSA. Proteasome inhibitors (MG132) and autophagy inhibitors (3-MA) are co-administered with CA. The details of the compounds are described in Table S1.

Reference

1. S. Spijker, in *Neuroproteomics,* K. W. Li, Ed. (Humana Press, Totowa, NJ, 2011), pp. 13-26.
